# A Multistage Fine-Tuning and Empathy Alignment Approach for an Emotional Support Dialogue System: Development and Evaluation Study

**DOI:** 10.2196/95603

**Published:** 2026-10-29

**Authors:** Xianbin Wang, Ruolin Wang, Wencheng Yang, Weijun Wang, Wei Pan, Min Jia, Shijie Gao, Xin Zhao

**Affiliations:** 1 School of Psychology, Central China Normal University, No.152 Luoyu Road, Hongshan District, Wuhan, Hubei, 430079, China, 86 15377637661; 2 School of Modern Information Industry, Guangzhou College of Commerce, Guangzhou, Guangdong, China; 3 BeiCheng Lvluo Institute for Social Psychological Intelligence Innovation and Development, Beijing City University, Beijing, China; 4 Manchester Institute of Education, The University of Manchester, Manchester, United Kingdom

**Keywords:** large language models, empathic dialogue systems, in-context learning, psychological counseling dataset, text-based emotional support, fine-tuning, generative AI, YuanChat

## Abstract

**Background:**

Large language models (LLMs) are increasingly being used for text-based emotional support. However, general-purpose LLMs may not consistently recognize emotional cues, adapt supportive strategies to contexts, or generate responses aligned with psychologically informed communication. The limited availability of large-scale, structured Chinese counseling data further constrains the development of models for context-sensitive emotional support.

**Objective:**

We developed and evaluated YuanChat, a family of specialized LLMs for context-sensitive emotional support; assessed whether E-URIAL improved response quality; and compared YuanChat with base LLMs.

**Methods:**

We constructed 2 datasets from anonymized real-world counseling sessions: ECD-3 (104,897 counselor response turns) for fine-tuning and *D_gold_* (9413 manually annotated counselor responses). Annotations covered emotional support conversation stages (exploration, comfort, and action), counseling techniques (clarification, support, and challenge), and empathic effects. We developed 2 YuanChat variants using GLM-4-9B-Chat and Huozi 3.5 as backbone models using parameter-efficient fine-tuning with low-rank adaptation (LoRA). We then developed E-URIAL, an inference-time framework combining predicted emotion and support strategy labels with retrieved labeled exemplars. Given the dialogue history through the help seeker’s latest utterance, each model generated a counselor-style response. We compared YuanChat with GPT-4, ERNIE 4.0, CharacterGLM, GLM-4-9B-Chat, and Huozi 3.5. The evaluation comprised (1) automatic evaluation on in-domain and external datasets, assessing empathy identification and empathic expression; (2) evaluation of empathy-related situational reasoning using an expert-developed multiple-choice benchmark; (3) evaluation by clinical experts (n=8) of empathic attunement, support strategy appropriateness, perceived helpfulness, and safety and professional boundary adherence; and (4) a complementary evaluation by volunteer master’s students in clinical and counseling psychology (n=50) of response fluency, empathy, and perceived helpfulness.

**Results:**

Fine-tuning increased YuanChat-Huozi3.5’s empathic expression score from 0.57 to 0.68; empathy identification score increased from 0.54 to 0.59. In expert evaluation, YuanChat-Huozi3.5 received a higher empathic attunement score than the base Huozi3.5 model (1.279 vs 0.327; mean difference 0.952, 95% CI 0.746‐1.152). Using professional counselor responses as the reference, the mean differences for YuanChat-Huozi3.5 were 0.148, 0.079, 0.039, and 0.002 in empathic attunement, supportive appropriateness, perceived helpfulness, and safety and professional boundary adherence, respectively. No statistically significant differences were detected between YuanChat-Huozi3.5 and the counselor references (all Holm-adjusted *P*≥.29). Higher scores were observed when E-URIAL was applied. When E-URIAL was applied with 3 retrieved exemplars, YuanChat-Huozi3.5 achieved an empathy identification score of 0.69 versus 0.59 without E-URIAL, an empathic expression score of 0.79 versus 0.68, and a perceived helpfulness score of 0.74 versus 0.61.

**Conclusions:**

The findings indicate that fine-tuning on real-world counseling data and annotating models with structured emotional support labels improved context-sensitive empathic responding in LLMs. Without updating model parameters, E-URIAL further improved empathy identification and empathic expression across multiple models. Together, these methods provide a scalable, theory-informed approach to enhancing the emotional support capabilities of LLMs.

## Introduction

### Background

The global mental health crisis has created an extensive demand for accessible counseling services; however, access to professional counseling remains limited, particularly in low- and middle-income countries [1,2]. Digital mental health technologies, particularly text-based empathic conversational agents, have emerged as potentially scalable approaches to narrowing the mental health service gap [3]. However, realizing the potential of these systems requires moving beyond generic expressions of comfort toward more context-sensitive and theoretically grounded emotional support [4].

Although modern large language models (LLMs) show strong general-purpose language capabilities, their empathic responses often remain repetitive, insufficiently probing, and inadequately attuned to the user’s specific emotional context [5-9]. Training LLMs on high-quality counseling dialogues grounded in established psychological frameworks can help them recognize context-specific emotional cues and select more appropriate support strategies [10].

Existing datasets, including EmpatheticDialogues and ESConv, have provided valuable benchmarks for developing and evaluating empathic dialogue systems [11-13]. However, their conversations were elicited from crowdworkers through recalled situations or role-play scenarios, rather than collected from naturally occurring interactions between help seekers and trained counselors (Table 1). Consequently, many existing datasets often lack ecological validity, offering a partial representation of the nuanced, multistage intervention strategies used by professional counselors, including those described in the helping skills framework [14]. This motivated the development of the Empathic Counseling Dialogue–3 (ECD-3) dataset, named for its 3 emotional support conversation stages: exploration, comfort, and action. On the basis of this, the *research question* (RQ) *1* was formulated:

*RQ1:* How does supervised fine-tuning (SFT) on a counseling-informed dataset affect the empathic performance of LLMs in emotional support dialogue?

**Table 1.** Empathic dialogue datasets.

Dataset	Data source	Construction method	Language^a^
EmpatheticDialogues (ED) [11]	Amazon Mechanical Turk	Crowdsourcing	English
SMILECHAT [15]	PsyQA dataset	LLM^b^-based multiturn synthesis	Chinese
Persona-based Empathetic Conversation [16]	Reddit	Rewritten from Reddit posts	English
Arabic EmpatheticDialogues [17]	Crowdsourcing	Translated from ED	Arabic
X-EMAC dataset [18]	User queries from XiaoAi online logs	Manual annotation	Chinese
EDOS dataset [19]	OpenSubtitles dataset	Automated extraction and generation	English
ESConv [12]	Crowdsourcing	Strategy-guided manual generation	English

a For datasets involving translation, the primary target language is reported to avoid ambiguity.

b LLM: large language model.

For LLMs’ empathic dialogue, fine-tuning matters because performance hinges on empathy language cues and strategy selection conditioned on the seeker’s affect [20]. To bridge this gap between general pretraining and specialized emotional support expertise, we used a fine-tuning strategy on the ECD-3 dataset. In particular, we used parameter-efficient fine-tuning techniques, such as weight-decomposed low-rank adaptation (DoRA) and quantized DoRA (QDoRA), for efficiently adapting the models while preserving their foundational reasoning capabilities. However, SFT alone encodes emotional support patterns in model parameters, leaving adaptation constrained by the training distribution and without dynamic access to case-specific guidance at inference. We therefore introduced empathy untuned LLMs with restyled in-context alignment (E-URIAL), a retrieval-augmented in-context learning (ICL) framework for inference-time alignment method that retrieves contextually relevant strategies and demonstrations to complement SFT and improve emotional support performance [21].

Conceptually, E-URIAL extends the untuned LLMs with restyled in-context alignment (URIAL) framework by incorporating theory-driven strategic constraints to guide empathic generation [22]. Drawing from psychological counseling literature, E-URIAL augments retrieved exemplars with multidimensional labels, including the emotional support conversation (ESC) stages, which conceptualize empathic support as a progression through exploration, comfort, and action, as well as specific counseling techniques and empathic language cues [12,14].

E-URIAL complements the parametric adaptation through a nonparametric mechanism. At inference time, it dynamically retrieves high-quality exemplars from the human-annotated subset and aligned with relevant support strategies. This hybrid approach combines the expertise encoded through fine-tuning with external, case-specific guidance, enabling more adaptive and context-sensitive emotional support responses without additional parameter updates. Thus, *RQ2* was formulated:

*RQ2*: How does E-URIAL affect task-specific empathic performance?

### Objectives

This study aimed to systematically enhance LLMs’ empathic response generation to provide more professional mental support in daily contexts. To address this, we define the following 3 specific research objectives:

- To construct a large-scale emotional support dataset (ECD-3) grounded in counseling knowledge and authentic counseling sessions;
- To enhance the emotional support capacity of LLMs by internalizing specialized knowledge through fine-tuning on the ECD-3 corpus; and
- To evaluate the effectiveness of E-URIAL, a retrieval-augmented framework, in providing dynamic strategic guidance for more adaptive and resonant mental support in everyday scenarios.

The primary contributions of this study can be organized into 3 dimensions:

- Data contribution: We introduce ECD-3, a large-scale counseling-dialogue dataset from China consisting of 104,897 counselor-response turns. Furthermore, we built *D_gold_*, a high-quality subset of 9413 counselor-response turns with multilayer labels, providing a structured annotation scheme informed by counseling theory. Model and evaluation contribution: We introduce YuanChat, a family of empathy-enhanced LLMs from China developed through fine-tuning on the ECD-3 dataset. Across automatic and human evaluations, we observe consistent improvements in human-rated empathy and support quality while maintaining strong linguistic fluency.
- Methodological contribution: We introduce E-URIAL, a retrieval-augmented ICL framework designed to steer empathic generation. By predicting strategy labels and dynamically retrieving exemplars, E-URIAL provides the model with professionally grounded guidance during inference. Our experiments suggest that this approach can improve empathic performance, offering a scalable solution for adaptive digital empathy.

### Related Studies

#### Empathic Capabilities of LLMs

Empathy is generally defined as the ability to recognize and respond to others’ emotional experiences, and it is particularly important in mental health support [23-25]. Prior studies have often distinguished cognitive empathy (understanding others’ emotions) from affective empathy (sharing or mirroring emotions) [26]. Recent studies have evaluated whether LLM outputs are perceived as empathic and whether they adapt to context (eg, [27,28]). Although many models can produce fluent supportive responses, they often show limited accuracy and preference bias in turn-level support strategy selection, reducing their ability to adapt to users’ changing emotional needs [29,30]. This limitation is particularly consequential in support-oriented, multiturn interactions. Examples include daily ESCs, chronic disease care, and educational tutoring. In such scenarios, empathic quality directly affects user trust and satisfaction [31-33].

A key reason is that pretraining on large-scale internet text exposes models to emotional language but provides limited specialized support knowledge for a systematic understanding of empathy [34]. SFT can yield incremental gains; yet, progress is often constrained by limited ecological validity and coarse supervision signals [35,36]. In mental health settings, agents perceived as more empathic are associated with higher user trust, highlighting the need for models that can move beyond generic consolation toward context-aware mental support [37-39].

#### Evaluation of LLMs’ Empathic Capabilities

Recent studies have evaluated empathy in LLMs using standardized benchmarks or psychometric instruments, automatic text-based metrics, human ratings, and hybrid protocols that combine automatic and human assessment [9,40-42]. As a benchmark study, EmoBench focuses on emotion understanding and response appropriateness under controlled scenarios, showing that models can approach human-level performance on some cognitive empathy tasks [39]. Human evaluation complements benchmarks by measuring perceived empathy. A previous study combined common automatic metrics (eg, Bilingual Evaluation Understudy [BLEU]-1 and BLEU-2 and embedding average cosine similarity) with human ratings and observed that models that incorporated an empathy recognition framework performed better on deep empathy tasks [41]. Despite these advances, current evaluation pipelines face 2 recurring limitations [43]. First, many schemes are weakly grounded in empathy support–related theories in annotation systems, leading to fragmented operationalizations, limited cross-model comparability, and reduced interpretability of evaluation outcomes [44]. Second, ecological validity is often limited because evaluation corpora and contexts cannot reflect authentic supportive interactions [43]. Such limitations motivate the need for theory-informed dataset construction and evaluation metrics aligned with professional constructs.

#### Enhancing LLMs’ Empathic Performance

##### Overview

On the basis of prior literature (Table 2), methods for improving empathic behavior can be broadly grouped into parameter updating approaches and inference-time approaches, or a combination of both [45,46]. Parameter updating methods such as SFT adapt model behavior using task-specific data, while inference-time methods (eg, ICL) steer generation with demonstrations without updating parameters [47].

**Table 2.** Prior studies.

Approach and dataset	Language	Primary technique
Parameter updating		
EmpatheticDialogues [11]	English	SFT^a^
SoulChatCorpus [20]	Chinese	SFT
ECC^b^ [48]	English	SFT
ServeForEmo [49]	English	Strategy-enhanced role-playing
KokoroChat [50]	Japanese	SFT
ESConv [12]	English	SFT
Inference time		
EmoBench [39]	English and Chinese	Zero shot and CoT^c^
EmpatheticDialogues [47]	English	Two-stage ICL^d^

a SFT: supervised fine-tuning.

b ECC: Emotion-Cause Conversation.

c CoT: chain-of-thought.

d ICL: in-context learning.

##### Parameter Updating Approaches

These methods improve empathic responses by training models on emotion-related corpora via SFT or preference-based optimization. Representative resources include EmpatheticDialogues and related emotion-annotated dialogue datasets. Strategy-oriented datasets such as ESConv offer finer supervision by annotating support strategies [51,52]. However, many existing corpora can exhibit limited realism for counseling-style interactions and sometimes lack explicit grounding in counseling theory. Domain-focused systems (eg, SoulChat) further suggest that adapting base models using mental support data can improve supportive response quality. Reinforcement learning from human feedback has been explored for empathy alignment, but it typically requires costly feedback and careful reward design [53-55].

##### Inference-Time Approaches

These methods guide generation at inference time using prompts or exemplars. Prior studies have explored role prompts and reasoning-oriented prompting for empathic responding [56]. Role-based prompts ask LLMs to respond as a specific helper role (eg, a patient listener or counselor), which can increase supportive and empathic language when compared with neutral prompts. For reasoning-oriented prompting, chain-of-thought prompting further encourages models to first reflect on the user’s emotional state before generating a response, improving contextual appropriateness. Emotion-conditioned prompting injects explicit affective cues into the prompt to improve emotion consistency [57], and a related study extended such prompting to spoken emotional understanding [58].

##### From URIAL to E-URIAL

The design of the prompts and the examples influences the effect of inference-time alignment methods. As a representative approach, the URIAL study suggested that base LLMs can achieve competitive alignment without parameter updates by combining a system prompt with 3 constant stylistic examples [22]. Subsequent studies have used URIAL as a baseline and examined adaptations of demonstration design and decoding settings. For example, in dynamic rewarding with prompt optimization, the study team used dynamic rewards to optimize alignment instructions without parameter updates [59].

For the approach design and dataset construction, grounding in established empathy and counseling frameworks provides theoretically valid and interpretable labels, distinguishes affective understanding from strategic intervention, and captures how professional support unfolds across dialogue stages. This structure converts counseling expertise into explicit supervision for model training, retrieval, and evaluation [60]. Thus, we integrated stage-wise support strategy and counseling practice in empathic dialogue and proposed E-URIAL, a counseling theory–informed extension of URIAL. E-URIAL extends URIAL in 2 key aspects (Figure 1).

1. Label-enriched exemplars: Each in-context exemplar is augmented with theory-informed labels, such as ESC-stage labels, counseling-technique labels [61], and empathic language cues, together with emotion information, so that exemplars can explicitly guide beyond semantic similarity.
2. Dynamic retrieval of in-domain counselor exemplars: Unlike URIAL, which uses fixed generic demonstrations, E-URIAL dynamically retrieves contextually relevant counselor exemplars. Candidate exemplars are ranked by sentence bidirectional encoder representations from transformers (SBERT)–based semantic similarity and incorporated, together with the predicted labels and system instruction, into the final prompt.

**Figure 1.**
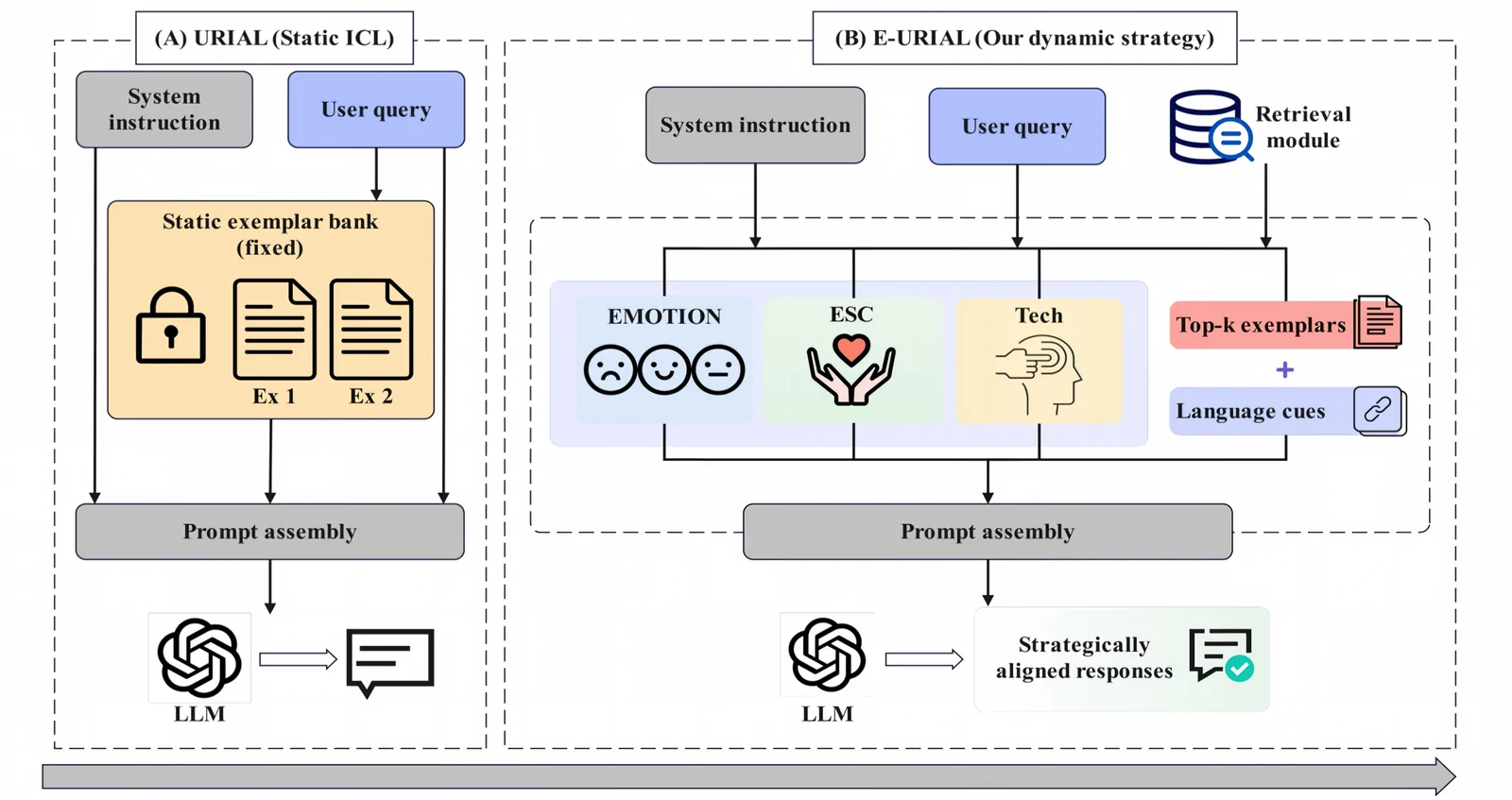
Prompt construction for URIAL (static in-context learning) and E-URIAL (dynamic strategy).

### Theoretical Foundations for Annotation, SFT, and Evaluation

We conceptualized emotional support response generation as 3 linked functions: recognizing the seeker’s state, selecting an appropriate support strategy, and realizing that strategy in language. Emotion- and context-related labels represent the seeker’s current state and support needs. ESC-stage labels locate the response within the broader support process, whereas counseling technique labels characterize the specific supportive techniques used in the response. We selected these variables because they correspond to the central task of this study: interpreting the seeker’s emotions and situation and generating a contextually appropriate supportive response.

At the process level, we adopted the ESC model, which builds on Hill’s helping skills theory and adapts helping strategies to everyday emotional support dialogue. The model organizes emotional support into exploration, comfort, and action. Exploration helps seekers articulate and clarify their situations, thoughts, and emotions. Comfort centers on empathy, understanding, and emotional support. Action helps seekers consider feasible ways to address their difficulties. These stages do not constitute a fixed sequence. Instead, the appropriate strategy depends on the seeker’s evolving needs and the dialogue context.

At the turn level, counseling techniques specify how a support strategy is enacted. We grouped these techniques into 3 categories [61]. Clarification techniques, such as asking for details, explaining concepts, and summarizing, help organize the seeker’s account and clarify the problem. Support techniques, including active listening, emotion reflection, and validation, communicate acceptance, understanding, and encouragement. Challenge techniques draw attention to discrepancies or potentially unhelpful patterns and invite the seeker to consider alternative interpretations or actions.

Together, the ESC stages and counseling techniques provide complementary process-level and turn-level structures for multilayer data annotation, SFT, and label-guided retrieval in E-URIAL. These constructs also informed the evaluation dimensions concerning state understanding, strategy appropriateness, empathy, and helpfulness.

### Research Overview

Building on the abovementioned review, we developed an integrated framework informed by empathic methodologies. First, we constructed a dataset from real counseling dialogues with deidentification and multilayer annotation. Second, we fine-tuned 2 base models on this dataset to obtain empathy-enhanced generation models. Third, to test whether retrieval can effectively guide generation, we introduced E-URIAL, which dynamically retrieves in-domain counseling exemplars to steer empathic generation. Finally, we evaluated both fine-tuned and inference-time methods using an assessment protocol grounded in the key elements of real counseling, thereby combining automatic metrics with human ratings. Figure 2 shows the overall study logic. The figure summarizes the main research logic from ECD-3 (our training data) dataset construction to supervised fine-tuning, E-URIAL–based retrieval-augmented in-context alignment, and evaluation. RQ1 encompasses dataset construction and supervised fine-tuning. RQ2 addresses E-URIAL–based retrieval-augmented alignment; manually annotated subset; and automatic evaluation metrics, which are empathy identification score, empathic expression score, and empathy ability calculation.

**Figure 2.**
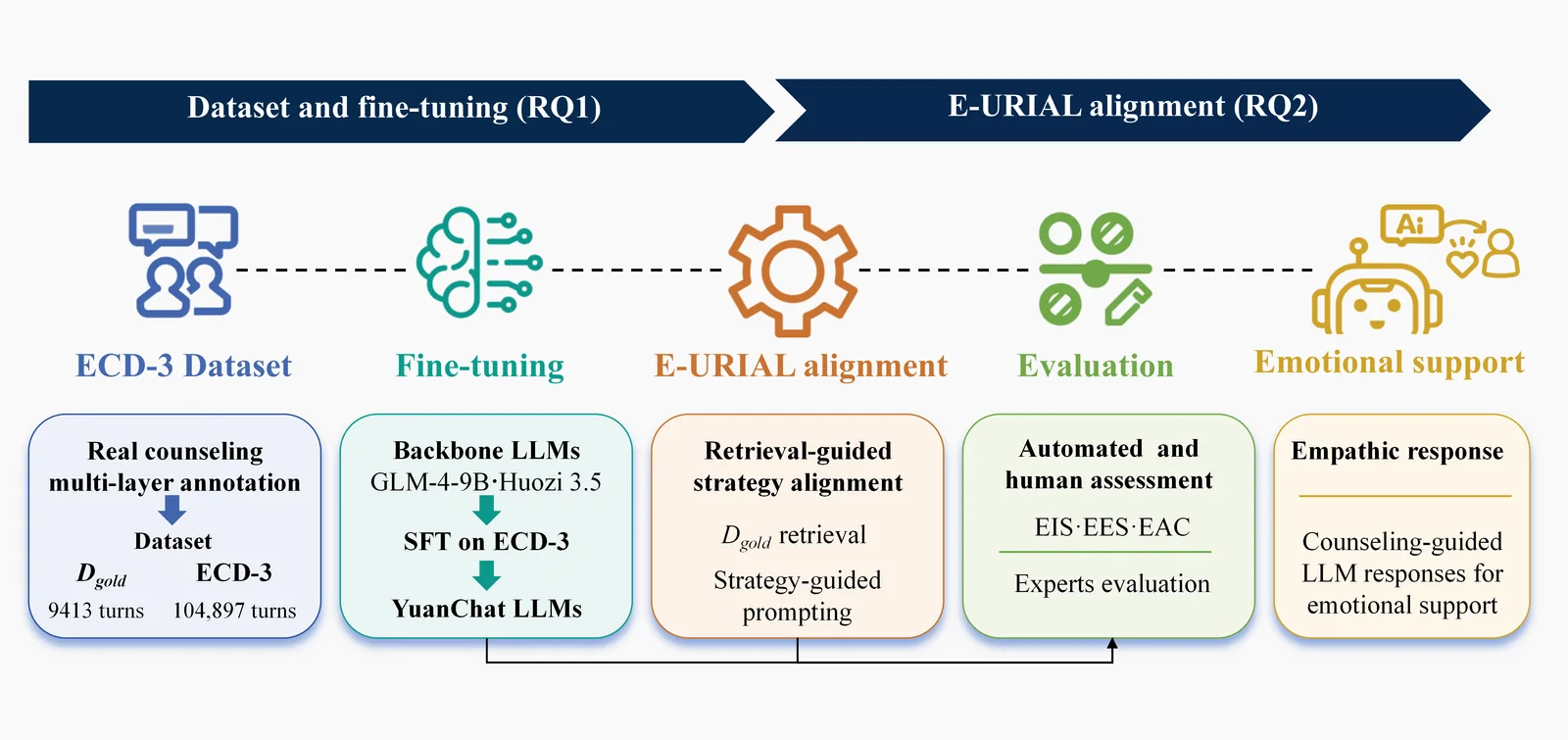
Overall study logic.

## Methods

### Study Design and Overview

This study followed a structured approach, encompassing 3 main stages: dataset creation, model development, and evaluation. In the first stage, we developed the 2 datasets: ECD-3 dataset and *D_gold_* dataset. ECD-3 contained 104,897 counselor-response turns. This dataset served as the foundation for the development of YuanChat models. *D_gold_* contained 9413 manually annotated counselor-response turns. This dataset was divided at the counseling case level into nonoverlapping evaluation and retrieval subsets. The evaluation subset was used to assess model performance. The retrieval subset provided counseling exemplars for E-URIAL. This study was prepared in accordance with the TRIPOD+LLM (Transparent Reporting of a Multivariable Prediction Model for Individual Prognosis or Diagnosis–Large Language Model) reporting guideline, and the completed checklist is provided in Checklist 1.

During model development, we separately fine-tuned GLM-4-9B-Chat and Huozi3.5 [62,63] on ECD-3, producing YuanChat-GLM4-9B and YuanChat-Huozi3.5. We also developed E-URIAL, an inference-time prompt enhancement framework that combined predicted emotion and support strategy labels with retrieved counselor exemplars. E-URIAL did not require additional training or parameter updates. To evaluate YuanChat’s performance, we conducted a comparative analysis with other generative AI models (eg, GPT-4 and CharacterGLM) and assessed its effectiveness through expert clinical assessments.

Model fine-tuning and analysis were performed using Python (Python Software Foundation) and Python-based machine learning frameworks and libraries. These included PyTorch (originally developed by Meta), DeepSpeed (originally developed by Microsoft), and Transformers, Datasets, and Accelerate (Hugging Face). The fine-tuning process leveraged high-performance computing resources to optimize model performance and efficiency. All local model training and inference were performed on a compute node equipped with 4 NVIDIA A100 80 GB graphics processing units (GPUs).

### Dataset Creation

#### Dataset Sources

The data were obtained from real-world counseling sessions conducted by a Chinese psychological service provider between May 2022 and May 2023. The corpus comprised sessions involving 68 counselors and 4063 clients. Among these clients, 3778 participated in audio-recorded sessions, whereas 285 used text-based counseling (Figure 3). The audio sessions lasted 30 to 90 minutes. We transcribed the audio recordings using Tencent Cloud’s automatic speech recognition API, which also performed speaker diarization. To accommodate the context window limits of foundation LLMs while preserving semantic coherence, we divided the dialogues into segments of no more than 1000 Chinese characters. We also removed nonsubstantive expressions, such as “En” and “Oo.” The processed dialogues were classified into 6 primary topics and an “Other” category, which included topics such as childhood trauma and cyberbullying (Figure 4). This preprocessing yielded 188,409 counselor response turns for subsequent screening and annotation.

The original corpus was divided into 2 separate parts for processing, which were used to construct the D_gold_ manually annotated dataset and the ECD-3 model training dataset. First, we selected 200 counseling cases with high satisfaction ratings for manual annotation. These cases comprised 9778 counselor response turns. Second, the remaining 178,631 counselor response turns were used as the candidate pool for construction of the ECD-3 training dataset.

**Figure 3.**
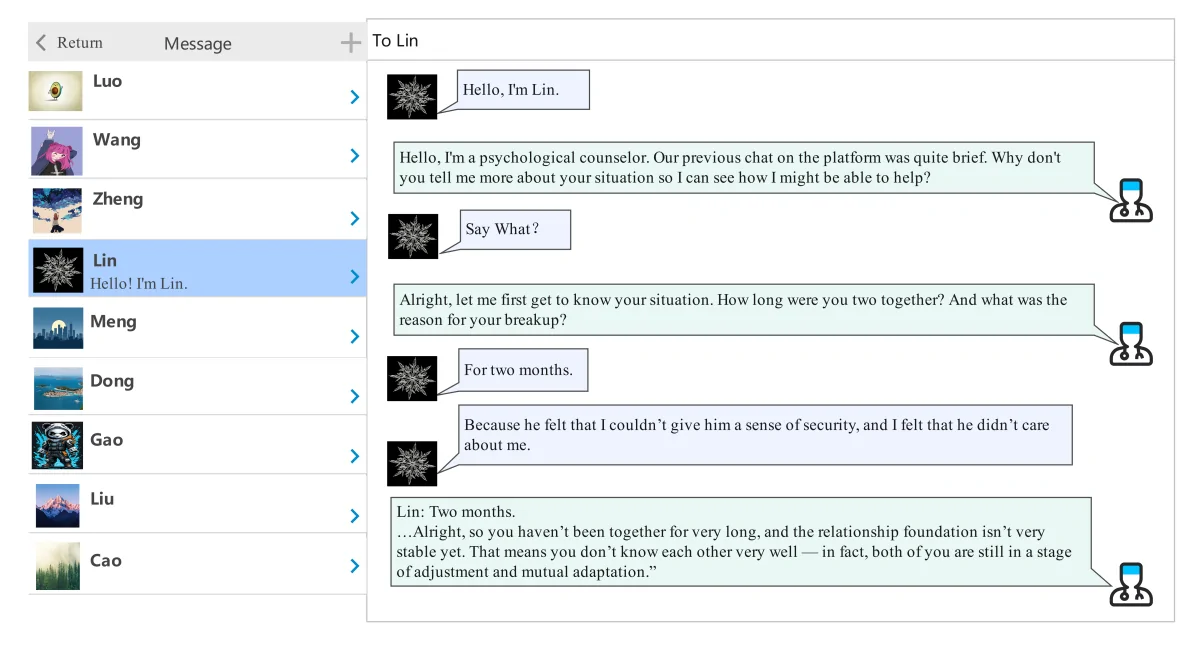
Example of an anonymized online counseling dialogue.

**Figure 4.**
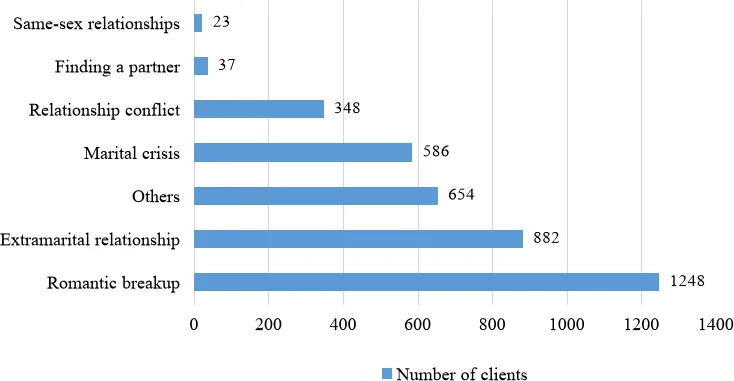
Number of clients by dialogue topic.

#### Annotation Guidelines

Annotation is a crucial stage in natural language processing studies that ensures the validity of psychological constructs [64]. We adopted a theory-driven annotation framework to map professional strategies to support empathic responses (Table 3).

**Table 3.** Annotation categories.

Category and subcategory	Assigned values
ESC^a^ stages
Exploration	0
Comfort	1
Action	2
Counseling techniques
Clarification	0
Support	1
Challenge	2
Empathy effect
Negative	−1
None	0
Weak	1
Strong	2

a ESC: emotional support conversation.

- ESC stages: On the basis of helping skills theory, this model divided emotional support into 3 stages, namely, exploration, comfort, and action, to support interactions in everyday social contexts.
- Counseling techniques: These were specific verbal interventions used for facilitating understanding and change. We annotated 3 core categories, namely, clarification, support, and challenge.
- Empathy effect: To ensure the inclusion of only high-quality empathic dialogue samples, we assessed the perceived quality of the empathy conveyed.

#### Annotation Process

We randomly recruited 8 data annotators from the authors’ university. All annotators were master’s students in psychological counseling and had at least 1 year of counseling practice experience. Before formal annotation, all annotators participated in a standardized training program that covered the annotation guidelines, representative examples, and live demonstrations. The training aligned their understanding and application of the psychological concepts underlying the annotation framework. All annotations were completed through a custom Streamlit platform. Figure 5 presents an example of the annotation process.

**Figure 5.**
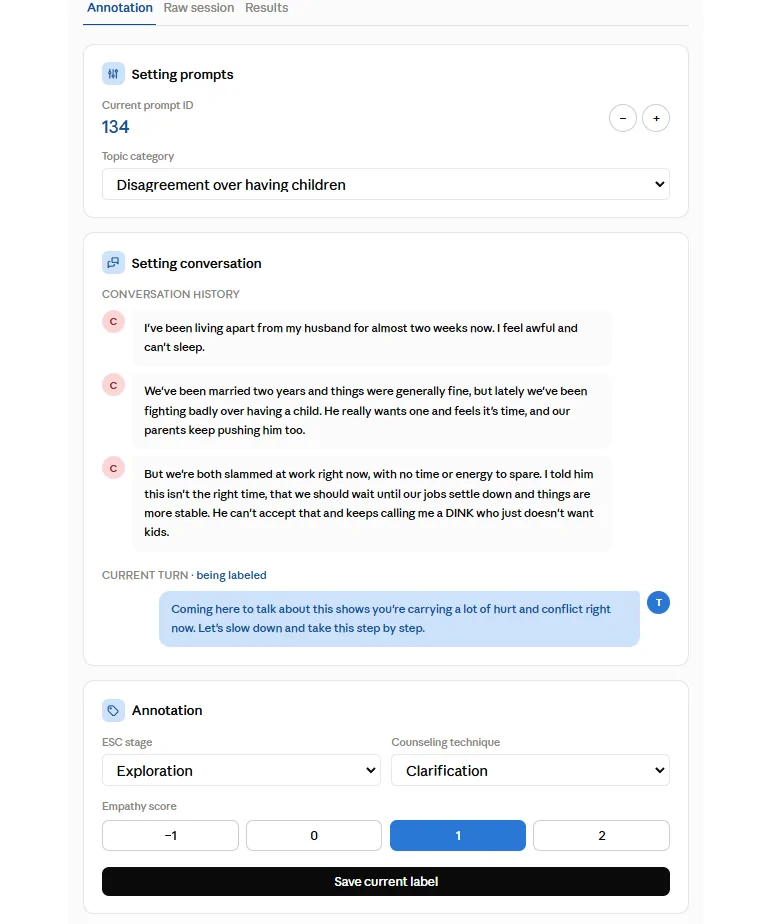
Annotation example.

We randomly selected 200 counseling cases. The annotators reviewed the dialogue context and labeled each counselor response in every selected case. For each assigned label, they also highlighted the relevant text spans in the response as evidence for the annotation, such as “*That is truly unfortunate*” and “*This is indeed distressing.*”

The initial annotation covered 9778 counselor responses to client utterances from 200 counseling cases. We excluded 19 cases comprising 365 responses because their labels were ambiguous or inconsistent, resulting in a final annotated dataset of 9413 counselor responses from 181 cases. We designated this manually annotated dataset as *D_gold_*. At the case level, we partitioned *D_gold_* into 2 subsets. The E-URIAL exemplar pool contained 7530 responses from 137 cases, whereas the held-out evaluation pool contained 1883 responses from 44 cases. None of the 44 evaluation cases appeared in either the ECD-3 training set or the E-URIAL exemplar pool, preventing case-level information leakage during model evaluation.

We assessed interrater reliability (IRR) using Cohen κ [64]. This coefficient is a chance-corrected measure suitable for categorical annotations, making it appropriate for our multidimensional annotation design [65]. Cohen κ was calculated for each annotator pair and averaged across pairs. The mean pairwise κ was 0.5857 for the ESC stages, 0.4219 for counseling techniques, and 0.5887 for empathy effect, indicating moderate agreement. We then reported the unweighted mean across dimensions as the overall IRR of 0.5321. The relatively low agreement on counseling techniques likely reflects the interpretive demands of fine-grained technique classification, a challenge also observed in prior psychotherapy annotation research [65].

#### Dataset Augmentation and ECD-3 Construction

Manual annotation was resource intensive and could not feasibly cover the full corpus [15]. We therefore constructed the ECD-3 from the 178,631 counselor response turns. We applied a dual-model label consistency procedure in which a locally deployed empathy strategy model and GPT-4 independently predicted the ESC stage and counseling technique labels for each response. A response was retained only when the 2 models assigned the same label for both annotation dimensions. This procedure retained 104,897 responses and excluded 73,734 responses because of label disagreement. GLM-4 subsequently rewrote each of the 104,897 retained responses under the prespecified constraints described below. This one-to-one rewriting procedure produced the final ECD-3 training dataset of 104,897 counselor response turns. Specifically, the ECD-3 dataset construction involved the following 3 steps.

#### Label-Consistency Filtering

We implemented a dual-model verification mechanism to ensure label reliability. A previously developed empathy computing model (Multimedia Appendix 1) and GPT-4 independently predicted the ESC stage and counseling technique associated with each counselor response. We retained a response only when the 2 models assigned the same label for both annotation dimensions. This criterion retained 104,897 responses and excluded 73,734 responses because of model disagreement.

#### Constrained Rewriting With GLM-4

We used GLM-4 (Zhipu AI) to rewrite the 104,897 retained responses. We selected GLM-4 because of its Chinese-language generation capabilities and support for a context window of up to 128,000 tokens [62]. Using the dialogue context and the agreed labels from step 1, GLM-4 rewrote each counselor response under three constraints: (1) strengthen the correspondence between the response and its assigned ESC stage and counseling technique, (2) preserve the original meaning and coherence of the dialogue context, and (3) check and remove any residual direct or indirect identifiers from the dialogue. The source counseling records had been anonymized before they were made available to the research team. As an additional safeguard, the rewritten outputs underwent a postrewriting privacy review, and any remaining potentially identifying information was removed before inclusion in ECD-3. This process produced 1 rewritten response for each retained response. The resulting ECD-3 dataset contained 104,897 counselor responses.

#### Expert Validation

To examine the quality of the ECD-3, we conducted an expert evaluation. We recruited 2 professionals with extensive experience in clinical psychology and psychological counseling through our professional networks. Using stratified random sampling, we selected 216 counselor response turns across 18 strata defined by 3 ESC stages and 6 dialogue topics, with 12 turns sampled from each stratum. The same-sex relationship topic was excluded because it contained too few eligible instances. Both experts independently evaluated all selected turns. Further details are provided in Multimedia Appendix 2.

On the basis of a previous framework [66], the evaluation was performed on a 5-point Likert scale across the following 3 crucial dimensions:

- Contextual authenticity: whether the response mirrored a real-world counseling scenario;
- Empathic appropriateness: whether the response aligned with professional empathy strategies and techniques; and
- Semantic naturalness: linguistic fluency and conversational flow of the rewritten text.

The results (Table 4) indicated that the sampled turns performed strongly on key dimensions. With a total score of ≥12 as the threshold for acceptance, the overall acceptance rate was 71.99%. IRR, assessed using Gwet AC2 [67], was 0.69 for contextual authenticity, 0.66 for empathic appropriateness, 0.68 for semantic naturalness, and 0.77 for the total score.

The results suggested that the generated responses were both context consistent and linguistically natural. Although empathic appropriateness was comparatively low, it remained above the midpoint of 3. A majority of the samples met the ≥4 criterion.

**Table 4.** Expert evaluation results.

Dimension	Mean (SD)	95% CI	Acceptance rate
Contextual authenticity	4.67 (0.43)	4.61-4.73	95.37% (≥4)
Empathic appropriateness	3.77 (0.91)	3.65-3.90	59.26% (≥4)
Semantic naturalness	4.18 (0.52)	4.11-4.25	77.31% (≥4)
Total score (sum of 3 dimensions; 3‐15)	12.63 (1.5)	12.42-12.84	71.99% (≥12)

### YuanChat Development

#### Datasets

We used ECD-3 exclusively for model fine-tuning. ECD-3 contained 104,897 counselor responses derived from real-world counseling sessions after label consistency filtering and constrained rewriting.

We evaluated the models using 2 test sources with distinct data provenance. The evaluation subset of *D_gold_* provided an in-domain benchmark comprising manually annotated counselor responses from real-world sessions. SMILECHAT served as an external source benchmark. It is a publicly available Chinese text-based dataset containing 55,165 multiturn mental health support dialogues. The dataset was constructed using the SMILE method, which prompted ChatGPT to transform publicly available single-turn question-answer pairs from PsyQA into dialogues containing at least five turns. The original study used SMILECHAT to examine lexical, semantic, and topic diversity and to fine-tune ChatGLM2-6B for developing the mental health support chatbot MeChat [15].

For the automatic evaluation, we randomly sampled 1000 counselor responses from the *D_gold_* evaluation pool and 1000 response instances from SMILECHAT, resulting in a balanced test set of 2000 instances. For the counseling trainee evaluation, we subsequently selected a stratified random sample of 100 dialogue contexts, including 50 instances from *D_gold_* and 50 from SMILECHAT. The clinical professional evaluation used the 70 real-world counseling contexts from *D_gold_*.

We also constructed an independent Chinese-language situational reasoning benchmark (EAC benchmark) comprising 200 multiple-choice items to evaluate empathy reasoning in LLMs. Each item presented a social or emotional scenario and required the model to infer the thoughts, feelings, or intentions of a person in that scenario. Each item contained 4 to 5 response options and 1 intended answer. Guided by the empathic accuracy framework and research on cognitive empathy and perspective taking [68-70], the items were developed by 5 licensed psychological counselors with a mean of more than 5 years of professional experience. The experts independently reviewed each item for construct relevance, clarity, cultural appropriateness, and answer ambiguity and independently identified the intended answer. Items without unanimous agreement were revised or excluded. Only items receiving unanimous agreement from all 5 experts after revision were retained. Examples are provided in Multimedia Appendix 3.

Table 5 summarizes the provenance, role, sampling procedure, and sample size of each dataset.

**Table 5.** Datasets and evaluation benchmarks.

Purpose	Dataset	Data provenance	Sampling	Instances, n
Model fine-tuning	ECD-3	Real-world counseling responses after label consistency filtering and constrained rewriting	All eligible responses	104,897
Automatic evaluation: in-domain	*D_gold_* evaluation subset	Manually annotated responses from real-world counseling sessions	Random sample	1000
Automatic evaluation: external source	SMILECHAT	ChatGPT-expanded Chinese multiturn mental health support dialogues	Random sample	1000
Automatic evaluation: situational reasoning	EAC^a^ benchmark	Expert-developed Chinese situational multiple-choice items	All eligible responses	200
Students in Clinical and Counseling Psychology evaluation	*D_gold_* and SMILECHAT	Subset of the automatic evaluation set	Random sample, 50 per source	100
Clinical professional evaluation	*D_gold_* evaluation subset	Real-world counseling contexts and authentic counselor responses	Random sample of *D_gold_* context	70

a EAC: Empathy Ability Calculation.

#### Models and Baselines

##### Overview

To comprehensively assess empathic performance, we compared our fine-tuned models against several baselines. For backbones, we selected 2 representative architectures for fine-tuning: GLM-4-9B-Chat and Huozi3.5 [62,63]. GLM-4-9B-Chat is a dense model optimized for long-context dialogue, following the GLM-4 family released by Zhipu AI and THUDM. Huozi3.5, released by HIT-SCIR, is a mixture-of-experts model further optimized based on HuoZi-3.0 and Chinese-Mixtral-8x7B.

For comparison models, we benchmarked against the following 3 strong proprietary models: GPT-4, CharacterGLM, and ERNIE 4.0. GPT-4 is the fourth generation of the GPT series [71]. It is trained on a very large and diverse text dataset and is well known for producing human-like responses. CharacterGLM was developed by Lingxin AI and Zhipu AI [72]. CharacterGLM is a derivative of ChatGLM optimized for “human-like” character interactions. ERNIE 4.0 is the fourth generation of Baidu’s ERNIE model [73]. ERNIE 4.0 worked well for complex, multiturn conversations and could adapt to different domains. All systems used the same prompt template. The specifications for all models are presented in Table 6.

**Table 6.** Specifications and roles of evaluated large language models.

Model	Role	Parameters (billion)	Layers	Model dimension (*d_*_model_)	Heads	Tokens
GPT-4	Baseline	—^a^	—	—	—	—
CharacterGLM	Baseline	6	28	4096	32	8000-32,000
ERNIE 4.0	Baseline	—	—	—	—	—
GLM-4-9B-Chat	Backbone	9	40	4096	32	128,000
Huozi3.5	Backbone	~46.7^b^	32	4096	32	32,000

a Not applicable.

b Mixture of experts model with 8 experts.

##### **SFT** Task and Data Formatting

The primary objective of our SFT was to enable models to generate expert-informed supportive responses. Given a seeker’s multiturn dialogue history and a system prompt, the model is trained to generate a coherent and professionally empathic reply.

We define a task as follows: Let X denote the multiturn dialogue context and L represent the expert-derived auxiliary labels (eg, ESC stage, counseling techniques, and emotion). The target is the supportive response $\text{Y}\text{ = (}{\text{y}}_{\text{1}}\text{,...}{\text{y}}_{\text{m}}\text{)}$. We formalize this as the following conditional generation problem:


$${\text{p}}_{\text{θ}}\text{(}\text{Y}\text{∣}\text{X}\text{,}\text{L}\text{)=}\prod_{\text{t}\text{=1}}^{\text{M}}{\text{p}}_{\text{θ}}\left({\text{y}}_{\text{t}}\text{∣}\text{X}\text{,}\text{L}\text{,}{\text{y}}_{\text{§amp;lt;}\text{t}}\right)$$

Training optimizes the token-level cross-entropy loss L(θ) using paired instances $\text{(}\text{X}\text{,}\text{L}\text{,}\text{and}{\text{Y}}^{\text{*}}\text{)}$:


$$L\left(\theta \right)=-\sum_{t=1}^{M}M_{t}\cdot \log p_{\theta }\left(y_{t}=y_{t}^{*}|X,L,y_{<t}\right),$$

where ${\text{M}}_{t}\in \{0,1\}$ denotes a loss mask ensuring that the gradient only updates on the basis of the assistant’s response, excluding the system prompt and labels. We fine-tune 2 decoder-only backbones, namely GLM-4-9B-Chat and Huozi3.5, separately.

##### Input and Output Formatting

To ensure compatibility with their distinct architectures, we use their respective native chat templates to concatenate multiturn dialogue histories. This alignment ensures that input formatting remains consistent with each model’s original training paradigm. We provide full template specifications in Multimedia Appendix 4.

The target output Y was the empathic reply to the final seeker query, designed to provide effective empathic support consistent with our human evaluation criteria. All training files were stored in the JSON format.

##### Training Setup

We used a learning rate of $1\times 10^{4}$, a per-device batch size of 4, and a maximum of 3000 training steps. The maximum input length was 2048 tokens, and the maximum output length was 2048 tokens. Training was conducted on 4 NVIDIA A100 GPUs (80 GB each).

### Implementation Details

#### Overview

We adopted parameter-efficient fine-tuning (PEFT) to adapt the backbones under a fixed compute budget: DoRA for GLM-4-9B-Chat and QDoRA for Huozi3.5.

DoRA decomposes pretrained weights into magnitude and direction components and fine-tunes them separately, while maintaining the same inference overhead as LoRA [74]. In our empathy-related task, this approach also improved instruction following. QDoRA (4-bit NF4 quantization with DoRA-based directional adaptation) injected adapters only into the activated expert subnetworks under Top-2 gating, enabling effective fine-tuning under limited GPU memory.

In both methods, the backbone model weights were frozen, and only the adapter parameters were trained. To ensure comparability, we controlled the maximum steps, per-device batch size (4), context length (4096 tokens), optimizer (8-bit AdamW), and learning rate schedule (cosine decay with 200 warmup steps) consistent across experiments. We also used the same prompt template and tokenization scheme in all settings. Our objective was to evaluate whether ECD-3 and E-URIAL improved empathic performance across representative backbones and not to compare PEFT algorithms. All experiments were conducted on a cluster equipped with 4 NVIDIA A100 80 GB GPUs, using DeepSpeed ZeRO-2/3 for memory optimization and FlashAttention-2 to accelerate attention computation. The core adapter hyperparameters for each backbone model, including rank, scaling factor, dropout, and quantization settings, are summarized in Multimedia Appendix 5.

#### E-URIAL Prompt Enhancement Framework

E-URIAL is an inference-time prompt enhancement approach adapted from URIAL for emotional support tasks. It requires no additional training or parameter updates. We evaluated whether its structured contextual guidance could improve emotional support responses across multiple LLMs and provide further gains for the domain-fine-tuned YuanChat models. As shown in Figure 6, for each input, E-URIAL first predicts emotion and empathy strategy labels and then retrieves the most semantically similar labeled counselor exemplars from the exemplar subset of *D_gold_*. The predicted labels, retrieved exemplars, system instruction, and current dialogue context are integrated into a structured prompt that guides the target LLM in generating the final supportive response.

**Figure 6.**
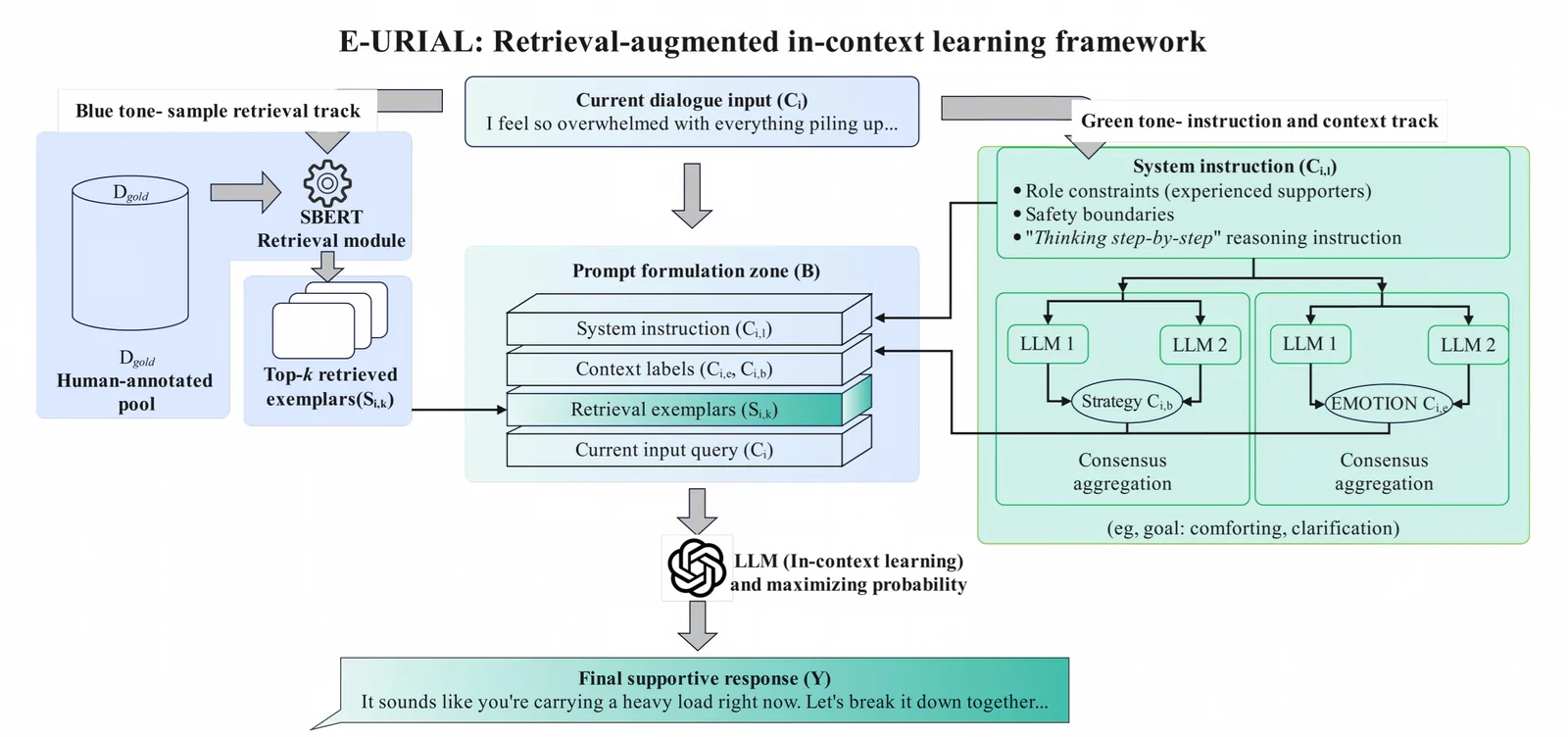
Empathy untuned large language models with restyled in-context alignment (E-URIAL) framework for retrieval-augmented in-context learning and prompt formulation**.** ${\text{C}}_{\text{i}}$: input; *D_gold_*: manually annotated dataset; EMOTION: inferred user emotional state; ESC: emotional support conversation stage; LLM: large language model; ${\text{S}}_{\text{i}\text{,}\text{k}}$: retrieved exemplars; SBERT: sentence bidirectional encoder representations from transformers; Y: output.

#### System Instruction

We specified the model’s role as an experienced psychological counselor who should respond professionally, empathically, honestly, and competently. The instruction also established safety boundaries prohibiting false, harmful, unethical, discriminatory, or otherwise socially irresponsible content and included a step-by-step reasoning instruction to promote structured and consistent responses.

#### Emotion Labels

For each input, we predicted 2 emotion labels for the help seeker: a dialogue-level emotion and a current-turn emotion. The dialogue-level emotion was inferred from the complete dialogue history up to the current turn and summarized the help seeker’s overall emotional state throughout the conversation. The current-turn emotion captured the immediate emotion expressed in the help seeker’s most recent utterance. We used a fixed YuanChat-Huozi3.5 checkpoint as the emotion-label predictor and applied a standardized prompt template to generate both labels: “Based on the dialogue history, the help seeker’s overall emotion is ...; in the current utterance, the help seeker’s emotion is ...” The predictions were mapped to Ekman’s 6 basic emotion categories: anger, disgust, fear, joy, sadness, and surprise. Cases that could not be reliably mapped to these categories were left unlabeled. To ensure comparability across response generation models, the emotion labels for each input were generated once and supplied unchanged to all evaluated models.

#### Composite Empathy Strategy Labels

We represented empathy strategy at 2 levels: strategic intent and linguistic realization. Strategic intent defined the appropriate supportive action. It comprised the ESC stage (exploration, comfort, or action) and counseling techniques (clarification, support, or challenge). Linguistic realization defined how the strategy was expressed. Empathic language cues guided the model to produce emotionally responsive and contextually appropriate wording.

For each input, we used a locally deployed strategy prediction model to predict the ESC-stage and counseling technique labels. Its architecture, training procedure, and validation results are described in Multimedia Appendix 1. After exemplar retrieval, we used a locally deployed empathic-language-cue tagging model to annotate the retrieved counselor exemplars. This specialized model used ChatGLM3-6B as its backbone and identified positive and negative empathy cues in each response. Its training procedure, validation results, and implementation settings are provided in Multimedia Appendix 6. Both labeling models remained frozen throughout E-URIAL inference. Their outputs were used only for prompt construction and were not used to train or update the response generation models. Neither labeling model had access to the target response or the corresponding counselor reference response from the evaluation set.

#### Sample Retrieval

We used SBERT to retrieve in-domain counselor exemplars from the retrieval subset of *D_gold_* (n=7530 responses). For each test input, SBERT encoded the dialogue context up to and including the help seeker’s most recent utterance. It also encoded the corresponding seeker-side context of each candidate exemplar. We applied mean pooling to the token representations to obtain a fixed-length sentence embedding for each context. Candidate exemplars were ranked according to their cosine similarity to the current dialogue context. The highest-ranked exemplars were retrieved together with their counselor responses and annotation labels and incorporated into the structured prompt.

The retrieval subset contained 7530 counselor response turns. It was separated from the evaluation subset at the counseling case level; therefore, no dialogue context or counselor reference response from the evaluation set was eligible for retrieval. To examine the effect of exemplar quantity under the input length constraints of the evaluated LLMs, we tested prompts containing 1, 3, and 10 retrieved exemplars.

#### Prompt Construction and Response Generation

We combined the system instruction, current dialogue context, predicted emotion and strategy labels, and retrieved labeled exemplars into a structured prompt. The resulting prompt was provided to the target LLM to generate the final supportive response.

#### Prompt Implementation Details

E-URIAL followed URIAL’s prefix prompt structure. The final prompt consisted sequentially of the system instruction, retrieved exemplars ranked by semantic similarity, and the current input. Each exemplar used standardized field markers to present the help seeker’s context, ESC stage, counseling technique, empathic language cues, and counselor response. The current input section contained the dialogue context up to the current turn and the predicted emotion, ESC stage, and counseling technique labels. The prompt ended with a response start marker, after which the target model generated the supportive response. After tokenization with the target model’s tokenizer, the maximum input length was set to 4096 tokens, and the maximum generation length was also set to 4096 tokens.

#### Evaluation

Building on prior research on emotional support dialogue, conventional metrics such as semantic similarity and general task performance do not adequately capture contextualized empathic expression. Existing studies therefore commonly evaluate models in terms of the appropriateness of empathic expression and their understanding of others’ emotions and perspectives. However, expert evaluations of professional appropriateness and safety remain limited [41-44]. Accordingly, our evaluation framework comprised 4 complementary components: response-level assessment of empathy identification and empathy expression, representing empathy strategy mechanism and empathy expression evaluation, respectively; assessment of empathy-related contextual understanding and reasoning; counseling trainee ratings of response quality; and clinical professional assessments of professional appropriateness and safety. As this study aimed to enhance emotional support capabilities rather than evaluate clinical effectiveness, symptom improvement and treatment outcomes were not included as evaluation variables.

Specifically, we used a 4-part evaluation framework to assess YuanChat and the comparison models. First, we conducted an automatic evaluation using test sets drawn from 2 different sources, *D_gold_* and SMILECHAT, to examine response quality of model performance across data sources. Second, we used scenario-based multiple-choice questions developed by experts to conduct an Empathy Ability Calculation (EAC) evaluation, testing the models’ ability to infer others’ emotions, perspectives, and intentions [75]. Third, counseling trainees conducted a blinded evaluation of the fluency, empathy, and perceived helpfulness of the model-generated responses. Fourth, clinical counseling and psychotherapy experts used real-world counselor responses as a professional baseline, assessing the model-generated responses for professional appropriateness, safety, and adherence to professional boundaries. By integrating automatic metrics, ratings from graduate students in clinical and counseling psychology, and professional judgments from counseling experts, this framework identified the strengths and limitations of each model in terms of performance across data sources, emotional support quality, and professional safety.

#### Automatic Evaluation

We used 2 automatic evaluation metrics: the Empathy Identification Score (EIS) and the Empathic Expression Score (EES).

##### Empathy Identification Score

EIS quantified the expressed empathy mechanism in model-generated responses using the EPITOME framework [44]. EPITOME captures both affective and cognitive components of empathy and evaluates each response in relation to the corresponding help seeker’s input, thereby supporting context-sensitive assessment of expressed empathy and making it suitable for the present study.

EPITOME characterizes textual empathy through 3 communication mechanisms: emotional reactions, interpretations, and explorations. Emotional reactions capture expressions of warmth, compassion, or concern in response to the seeker’s experiences. Interpretations capture communicated understanding of those experiences. Explorations capture gentle and specific inquiries into feelings or experiences that the seeker has not explicitly stated. EPITOME uses a multitask biencoder architecture built on RoBERTa-base. The S-Encoder encodes the help seeker’s input, whereas the R-Encoder encodes the corresponding response. An attention mechanism integrates the 2 representations to identify empathic expression in the response. The released model includes 3 independently trained classifiers for emotional reactions (ER), interpretations (IP), and explorations (EX). Each classifier assigns the corresponding mechanism an expression level of 0, 1, or 2, representing no, weak, or strong expression, respectively.

We adapted the publicly released EPITOME model for Chinese mental health support dialogues and internally validated the resulting evaluator. On the test set drawn from *D*_*gold*_, its accuracy and macro-*F*_1_-score were 0.8875 and 0.7457 for ER, 0.8599 and 0.7468 for IP, and 0.8850 and 0.7189 for EX. Details of its Chinese adaptation and training are reported in Multimedia Appendix 7.

For each response, we summed the predicted expression levels for ER, IP, and EX and divided the result by the maximum possible total of 6, producing a normalized response-level EIS ranging from 0 to 1. We then calculated the EIS for each evaluated model by averaging its response-level scores across all responses. Higher EIS values indicated greater expression of the 3 empathy mechanisms.

##### Empathic Expression Score

As strategy alignment does not necessarily ensure that support is linguistically expressed in an emotionally validating and context-sensitive manner, EES was included to assess the realized empathic expression in each response. EES was treated as a supplementary proxy measure and was interpreted alongside the blinded ratings provided by counseling trainees and clinical professionals. EES assessed the extent to which a generated response conveyed contextually appropriate emotional understanding and supportive expression. Responses were evaluated using a prespecified rubric covering emotional recognition, perspective understanding, and supportive communication. For scalable automatic scoring, a separate LLM evaluator applied the same rubric to all responses. To assess scoring accuracy, the researchers randomly sampled 200 of the responses and manually checked the automated cue labels against the prespecified rubric. Following the cue-coding procedure detailed in Multimedia Appendix 6, the evaluator identified the presence of prespecified positive empathy cues (P) and negative cues (N).

For the ith response, $h_{i,j}\in \{0,1\}$ denoted whether empathy cue J was present, with 1 indicating presence and 0 indicating absence. Cue presence was identified by the rubric-based automatic evaluator. Each cue was assigned a weight according to its polarity: ${\text{w}}_{\text{+}}$ for positive cues and ${\text{w}}_{\text{-}}$ for negative cues. The default weights were ${\text{w}}_{\text{+}}\text{ =1}$ and ${\text{w}}_{\text{-}}\text{=-1 }$. EES was calculated as follows:


$${\text{EES}}_{\text{i}}\text{=}\sum_{\text{j}\text{=1}}^{\text{J}}{\text{w}}_{\text{j}}{\text{h}}_{\text{ij}}\text{,}$$

where


$${\text{w}}_{j}=\left\{ \begin{array}{cc} w_{+}, & if\;j\;is\;a\;\text{positive}\;empathy\;cue,\\ w_{-}, & if\;j\;is\;a\;negative\;empathy\;cue. \end{array}\right.$$

$$\text{EES=}\frac{\text{1}}{\text{N}}\sum_{\text{i}\text{=1}}^{\text{N}}{\text{EES}}_{\text{i}}\text{,}$$

where J=25 denotes the number of prespecified empathy cues, and N denotes the number of evaluated responses. The complete inventory and polarity classification of the 25 linguistic cues are provided in Multimedia Appendix 8. Higher EES values indicate a greater presence of positive empathic expressions relative to negative expressions.

##### Empathy Ability Calculation

As empathic wording may reflect stylistic imitation without understanding of another person’s internal state, EAC was included to assess empathy-related situational reasoning independently of response fluency and supportive language use. EAC assessed empathy-related situational reasoning, defined as the ability to infer another person’s thoughts, feelings, and intentions from contextual cues. EAC was calculated as the proportion of correctly answered items in the 200-item expert-developed situational reasoning benchmark described earlier:

 .$$\text{EAC=}\frac{\text{Number}\text{of}\text{correctly}\text{answered}\text{items}}{\text{200}}$$

Higher EAC scores indicated greater accuracy in empathy-related situational reasoning.

### Human Evaluation

For a more comprehensive evaluation of YuanChat and E-URIAL, we subsequently conducted a human evaluation. We recruited 50 master’s students from the authors’ university. Participants were recruited through public notices posted on campus bulletin boards and social media. All participants joined voluntarily and received no compensation. Eligibility criteria required participants to be master’s students in clinical or counseling psychology and to have completed more than 50 hours of formal training in counseling skills. The participants comprised 27 women and 23 men, ranged in age from 23 to 25 years, and had a mean age of 24.8 (SD 0.49) years. Before participation, we obtained written informed consent and informed the participants that the data would be used for research purposes. All participants were required to read the evaluation instructions, operational definitions of each dimension, and scoring examples. No private or personally identifiable information was collected during the study.

We sent the evaluation forms to the participants by email. The evaluation set comprised responses generated by 7 models for 100 dialogue contexts randomly sampled from *D_gold_* and SMILECHAT. We used a randomized balanced assignment design. Each evaluator assessed 10 dialogue contexts and rated the 7 anonymized model-generated responses for each context, resulting in 70 context-response pairs per evaluator. Each dialogue context was independently evaluated by 5 evaluators; therefore, each model-generated response received 5 independent ratings. To reduce order effects, the presentation order of the 7 responses within each context was randomized. Model identity and dataset source were concealed from the evaluators.

Evaluators rated each response on a 3-point scale (0=poor, 1=fair, and 2=good) across the following dimensions:

- Fluency (Flu.) assessed grammatical correctness, coherence, readability, and naturalness. A score of 0 indicated errors that substantially impeded comprehension; 1 indicated an understandable response with minor errors or awkward wording; and 2 indicated a grammatically correct, coherent, and natural response.
- Empathy (Emp.) assessed whether the response appropriately recognized and addressed the seeker’s emotions and perspective. A score of 0 indicated that the response ignored, misinterpreted, or responded inappropriately to the seeker’s emotional state; 1 indicated partial or generic emotional recognition; and 2 indicated accurate, sensitive, and contextually appropriate emotional understanding.
- Perceived helpfulness (Help.) assessed the perceived usefulness of the response in providing emotional support. A score of 0 indicated an irrelevant, unhelpful, or potentially inappropriate response; 1 indicated a somewhat relevant but generic or limited response; and 2 indicated a relevant, constructive, and potentially helpful response. This dimension assessed perceived emotional support rather than symptom improvement, therapeutic efficacy, or clinical outcomes.

Interrater agreement was assessed using Fleiss κ. The coefficients were 0.69 for fluency, 0.77 for empathy, and 0.73 for perceived helpfulness.

### Evaluation by Clinical Experts

To evaluate the effect of fine-tuning with ECD-3 on the models’ emotional support performance, we recruited 8 clinical professionals with direct experience in clinical and counseling psychotherapy through our professional networks and academic connections. The panel included 2 recent clinical counseling graduates who were working in hospital psychology outpatient clinics; 3 certified psychotherapists with at least 3 years of professional experience in clinical psychology; and 3 psychologists registered with the Clinical and Counseling Psychology Registration System of the Chinese Psychological Society, each with at least 5 years of experience in clinical psychological counseling and psychotherapy. All evaluators participated voluntarily and received no financial compensation.

We used 70 real counseling scenarios from *D_gold_*. For each context, the evaluators were presented with responses generated by 6 high-performing models (CharacterGLM, GPT-4, GLM-4-9B-Chat, YuanChat-GLM4-9B, Huozi3.5, and YuanChat-Huozi3.5) and the original response of the counselor in the same counseling session, yielding 490 context-response pairs. All responses were anonymized.

We developed a dedicated web-based platform for the expert evaluation (Figure 7). Evaluators first provided written informed consent and then accessed an evaluation manual within the platform. The manual described the evaluation procedure, the operational definitions of the 4 dimensions, and the criteria for assigning each score. After completing standardized training, evaluators reviewed each dialogue context and rated each response on a 3-point scale (0=poor, 1=fair, and 2=good). The 4 dimensions were defined as follows.

Empathic attunement assessed how accurately and sensitively the response recognized and addressed the seeker’s emotions, circumstances, and perspective. Appropriateness of the support strategy assessed whether the response applied an appropriate ESC strategy while remaining relevant, respectful, nonjudgmental, and suited to the help seeker’s needs and conversational context. Perceived helpfulness assessed the response’s potential to provide meaningful emotional support or an appropriate next step. Safety and adherence to professional boundaries assessed whether the response avoided potentially harmful content, unsupported diagnoses, and unwarranted treatment recommendations or assurances. It also considered appropriate acknowledgment of risk and referral when warranted. After rating all responses within a dialogue context, evaluators selected the response they judged best overall, considering all 4 dimensions.

**Figure 7.**
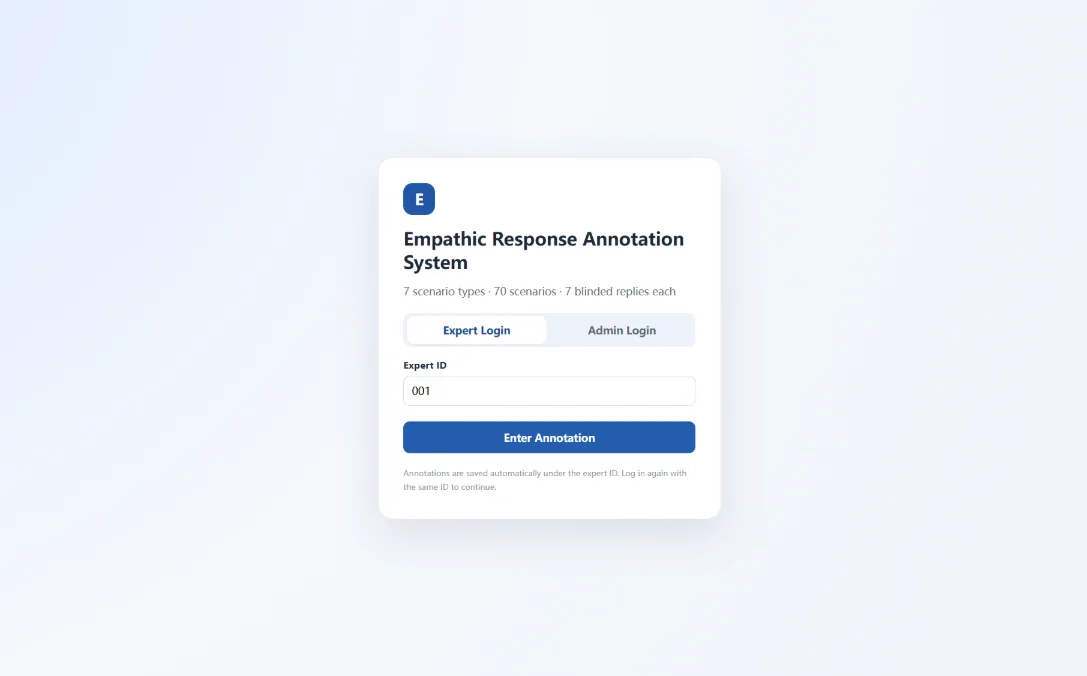
Expert evaluation platform.

### Ethical Considerations

The collection and secondary research use of the counseling records used to construct ECD-3 and *D*_*gold*_ were approved by the Ethics Committee of Huazhi Xinzhu Technology (approval HZXZ-IRB-202201017). Before receiving counseling services, each service user completed a written informed consent form that included an option to permit the use of their anonymized counseling records for research purposes. Only records from users who explicitly consented to such use were eligible for inclusion in ECD-3 and *D*_*gold*_; records from users who did not provide consent were excluded. The data were anonymized before being provided to the research team, and no personally identifiable information was collected or received by the researchers.

The human evaluation procedures were approved by the Institutional Review Board of Central China Normal University (approval CCNU-IRB-202403051). All evaluators provided written informed consent before participation. Participation was entirely voluntary and anonymous, and no compensation was provided for participation in this study. No personally identifiable information was included in the evaluation data analyzed in this study.

## Results

This section presents the empirical findings of our study, which are organized into the following 3 parts: SFT performance (RQ1), efficacy of the E-URIAL framework (RQ2), and the ablation study validating our framework design.

### Performance of Fine-Tuned Models (RQ1)

Table 7 presents the performance comparison between foundation models and our fine-tuned variants on the *D_gold_* and SMILECHAT datasets. Table 8 presents the empathy reasoning performance for each model. Overall, fine-tuning on the ECD-3 dataset yielded consistent improvements across empathy-specific metrics.

**Table 7.** Automatic evaluation results^a^.

Input dataset and model	EIS^b^, mean	EES^c^, mean	Human assessment, mean		
			Flu.^d^	Emp.^e^	Help.^f^
*D*_*gold*_					
ERNIE 4.0	0.41	0.41	0.91	0.72	0.48
CharacterGLM	0.65	0.75	0.91	0.85	0.68
GPT-4	0.45	0.54	0.95	0.76	0.55
GLM-4-9B-Chat	0.40	0.38	0.84	0.61	0.40
YuanChat-GLM4-9B	0.50	0.60	0.89	0.79	0.61
Huozi3.5	0.54	0.57	0.88	0.76	0.67
YuanChat-Huozi3.5	0.59	0.68	0.93	0.86	0.62
SMILECHAT					
ERNIE 4.0	0.47	0.57	0.94	0.74	0.56
CharacterGLM	0.63	0.71	0.92	0.89	0.73
GPT-4	0.45	0.54	0.96	0.79	0.53
GLM-4-9B-Chat	0.42	0.41	0.87	0.72	0.62
YuanChat-GLM4-9B	0.53	0.67	0.88	0.81	0.62
Huozi3.5	0.49	0.55	0.87	0.74	0.65
YuanChat-Huozi3.5	0.59	0.61	0.87	0.83	0.67

a Automatic evaluation was conducted using 1000 response instances from each dataset. Counseling trainee ratings were based on 50 dialogue contexts from each dataset.

b EIS: Empathy Identification Score.

c EES: Empathic Expression Score.

d Flu.: fluency

e Emp.: empathy.

f Help.: Perceived helpfulness.

**Table 8.** Empathy-related situational reasoning results.

Model	Correct responses (N=200), n	EAC^a^
ERNIE 4.0	148	0.74
CharacterGLM	140	0.70
GPT-4	158	0.79
GLM-4-9B-Chat	116	0.58
YuanChat-GLM4-9B	126	0.63
Huozi3.5	144	0.72
YuanChat-Huozi3.5	146	0.73

a EAC indicates Empathy Ability Calculation and was defined as the proportion of correctly answered items on the independent 200-item situational reasoning benchmark.

#### Huozi3.5 Versus YuanChat-Huozi3.5

The fine-tuned Mixtral model achieved higher scores than its corresponding baseline on *D_gold_*. It presented improvement in EIS (+0.05) and EES (+0.11), suggesting that task-specific fine-tuning aligned the model’s output with professional counseling standards.

On the out-of-domain SMILECHAT dataset, both fine-tuned models generally achieved higher scores than their corresponding base models. Compared with GLM-4-9B-Chat, YuanChat-GLM4-9B achieved higher EIS (0.53 vs 0.42), EES (0.67 vs 0.41), and empathy ratings (0.81 vs 0.72), while its fluency rating increased slightly and perceived helpfulness remained unchanged. YuanChat-Huozi3.5 also achieved higher scores than Huozi3.5 in EIS (0.59 vs 0.49), EES (0.61 vs 0.55), empathy (0.83 vs 0.74), and perceived helpfulness (0.67 vs 0.65), with no change in fluency. These results indicate that the performance gains associated with fine-tuning on ECD-3 extended to the external dataset, particularly for empathy-related measures. These findings suggest that the benefits of fine-tuning on ECD-3 generalized to a dataset differing in source and dialogue characteristics, primarily improving empathy-related performance without compromising fluency.

#### GLM-4-9B-Chat Versus YuanChat-GLM4-9B

The fine-tuned variant achieved considerably higher scores than its base model across all empathy dimensions. Notably, EES (empathic language cues) increased from 0.38 to 0.60, and EAC (empathic reasoning) improved from 0.58 to 0.63. Human evaluators rated YuanChat-GLM4-9B higher in perceived empathy (Emp: 0.79 vs 0.61), validating that even smaller parameters (9B) could lead to substantial empathy gains when aligned with high-quality domain data.

These results addressed RQ1 by suggesting that fine-tuning on ECD-3 improved emotional support performance in both model families. Across *D_gold_* and SMILECHAT, YuanChat-GLM4-9B and YuanChat-Huozi3.5 achieved higher EIS, EES, and human-rated empathy scores than their corresponding base models. The same pattern across the 2 datasets suggests that the improvements in empathy-related performance were not limited to the in-domain evaluation data.

The cross-model comparisons also distinguished linguistic fluency from empathic performance. GPT-4 achieved the highest fluency scores on both datasets (0.95 and 0.96), but its empathy ratings were lower than those of both fine-tuned models. YuanChat-GLM4-9B and YuanChat-Huozi3.5 also exceeded GPT-4 in EIS and EES, although CharacterGLM obtained the highest overall empathy-related scores. These results indicate that high linguistic fluency alone does not ensure strong empathic performance and that task-specific fine-tuning contributed to better performance on the evaluated empathy measures. As the models differed in architecture, parameter scale, and pretraining, the present comparisons do not isolate the effect of model size.

### Expert Evaluation Results

We summarized the ratings provided by 8 experts across 4 dimensions: empathic attunement, supportive appropriateness, perceived helpfulness, and safety and professional boundary adherence. Each expert rated all 490 responses, representing 7 response sources across 70 scenarios. We first calculated descriptive statistics. Figure 8 presents the mean expert ratings and corresponding 95% CIs for each dimension. We then used 2-sided Wilcoxon signed-rank tests to compare each model with the corresponding human counselor response from the same scenario. Holm correction was applied to the 6 model-counselor comparisons within each rating dimension. Differences were defined as model minus counselor, and 95% CIs for the mean differences were obtained from 100,000 bootstrap resamples using the dialogue scenario as the resampling unit. Interrater agreement was assessed using ordinal Krippendorff α for each rating dimension. All analyses were conducted using Python 3.12.13, NumPy 2.3.5, and pandas 3.0.1.

**Figure 8.**
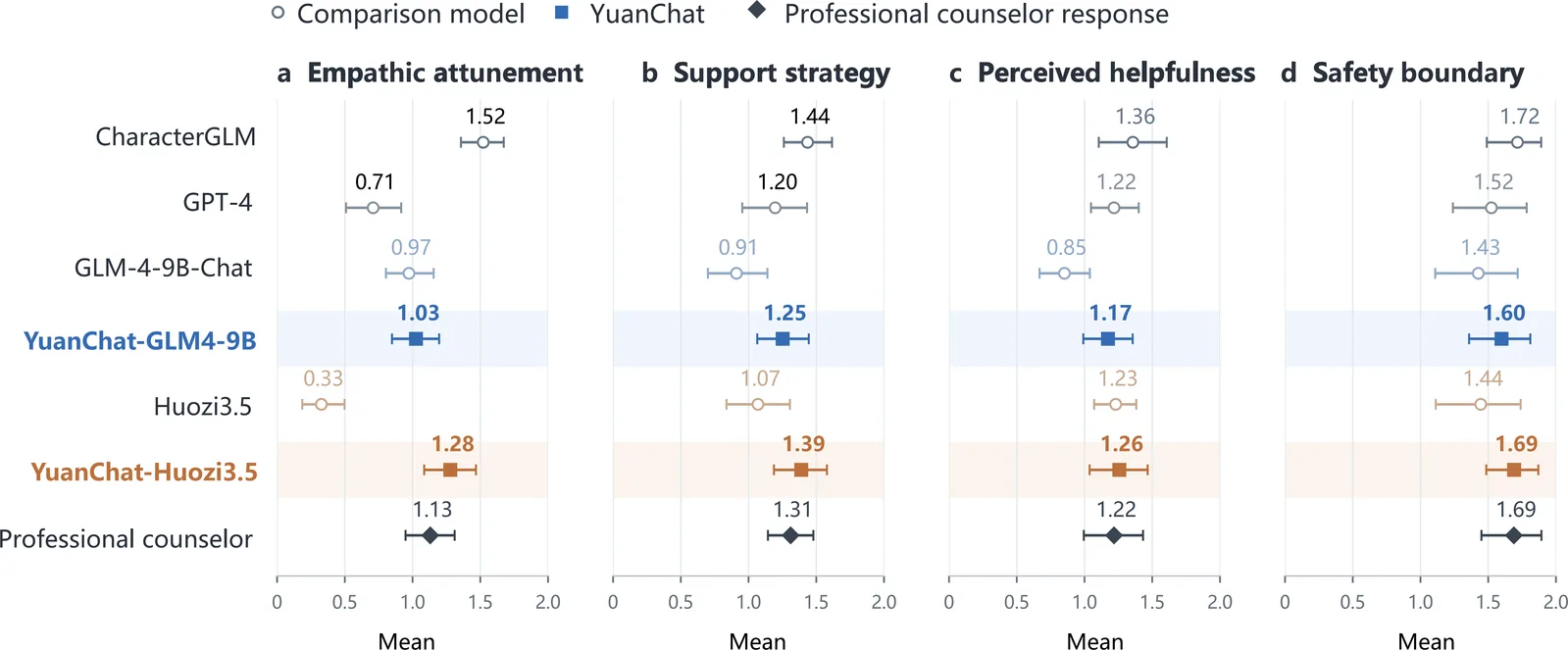
Mean expert ratings and 95% CIs. Points indicate mean ratings, and horizontal lines indicate 95% CIs. Responses from the 7 sources were grouped into 3 categories, as indicated in the legend: comparison models, YuanChat models, and professional counselor responses. GLM: general language model; GLM-4: general language model, 4th generation; GLM-4-9B: general language model, 4th generation, 9 billion parameters; GPT-4: generative pretrained transformer 4.

From descriptive results (Table 9), compared with Huozi3.5, YuanChat-Huozi3.5 received higher empathic attunement ratings (1.279 vs 0.327; mean difference 0.952, 95% CI 0.746-1.152) and supportive appropriateness ratings (1.389 vs 1.070; mean difference 0.320, 95% CI 0.134-0.502). Compared with GLM-4-9B-Chat, YuanChat-GLM4-9B received higher supportive appropriateness ratings (1.252 vs 0.911; mean difference 0.341, 95% CI 0.209-0.470) and perceived helpfulness ratings (1.173 vs 0.852; mean difference 0.321, 95% CI 0.102-0.541). CharacterGLM had the highest observed mean rating in each dimension, followed by YuanChat-Huozi3.5. The Krippendorff α was 0.43 for empathic attunement, 0.51 for supportive appropriateness, 0.55 for perceived helpfulness, and 0.49 for safety and professional boundaries.

**Table 9.** Descriptive results of expert evaluation.

Response source	Empathic attunement, mean (SD)	Supportive strategy, mean (SD)	Perceived helpfulness, mean (SD)	Safety and professional boundaries, mean (SD)
CharacterGLM	1.521 (0.567)	1.436 (0.554)	1.357 (0.625)	1.716 (0.486)
GPT-4	0.709 (0.687)	1.196 (0.613)	1.218 (0.646)	1.523 (0.615)
GLM-4-9B-Chat	0.973 (0.675)	0.911 (0.679)	0.852 (0.746)	1.427 (0.648)
YuanChat-GLM-4-9B	1.025 (0.739)	1.252 (0.603)	1.173 (0.658)	1.598 (0.539)
Huozi3.5	0.327 (0.579)	1.070 (0.690)	1.230 (0.697)	1.445 (0.653)
YuanChat-Huozi3.5	1.279 (0.728)	1.389 (0.615)	1.257 (0.701)	1.691 (0.492)
Professional counselor	1.130 (0.779)	1.311 (0.652)	1.218 (0.689)	1.689 (0.493)

### Model Performance Relative to Professional Counselor Responses

Using responses from professional counselors as the baseline, we compared the performance differences of each model across 4 evaluation dimensions. Wilcoxon signed-rank tests were conducted using paired mean scores from 70 scenarios. Exact zero differences were excluded, and 2-sided asymptotic *P* values were adjusted for tied ranks. Holm correction was applied to the 6 model-counselor comparisons within each rating dimension. shows that YuanChat-Huozi3.5 was the only model for which the comparisons with counselor responses did not reach statistical significance in any of the 4 dimensions; the remaining models all exhibited significant gaps compared to counselors in various dimensions. Specifically, the mean differences between YuanChat-Huozi3.5 and the counselor responses were 0.148 for empathic attunement, 0.079 for supportive appropriateness, 0.039 for perceived helpfulness, and 0.002 for safety and professional boundary adherence. All 95% CIs included 0, and the Holm-adjusted *P* values were ≥.29. In contrast, the base Huozi3.5 model received significantly lower ratings than the counselor responses for all dimensions.

**Figure 9. F9:**
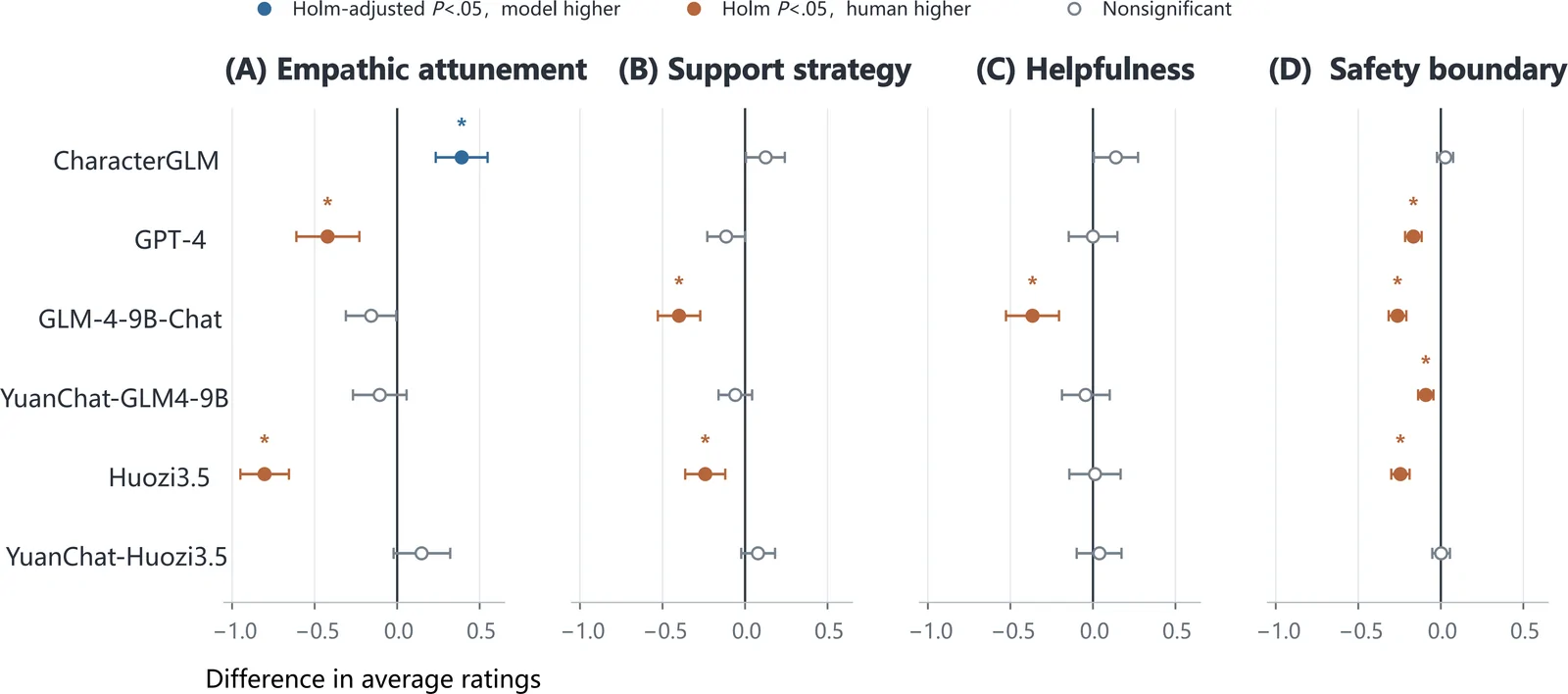
Differences in ratings relative to professional counselor responses. Asterisks and filled markers indicate Holm-adjusted *P*<.05.[Fig F9]

The other models showed dimension-specific differences. GPT-4 received lower ratings for empathic attunement and safety and professional boundary adherence; GLM4-9B-Chat received lower ratings for supportive appropriateness, perceived helpfulness, and safety and professional boundary adherence; YuanChat-GLM4-9B received lower ratings for safety and professional boundary adherence; and CharacterGLM received higher empathic attunement ratings than the counselor responses. Complete results are presented in Table 10.

Across all 4 dimensions, YuanChat-Huozi3.5 received expert ratings close to those of the professional counselor responses. This pattern suggests that training tailored to psychological support scenarios brought the model’s expert-rated performance closer to the professional counselor baseline in empathic attunement, supportive appropriateness, perceived helpfulness, and safety and professional boundary adherence.

**Table 10.** Paired comparisons of 6 model responses with professional counselor responses^a^.

Rating dimension (counselor mean)	Model (model mean)	Model − counselor, mean difference (95% CI)	Wilcoxon W	Holm-adjusted *P* value
Empathic attunement (1.130)	YuanChat-Huozi3.5 (1.279)	+0.148 (−0.021 to 0.321)	877.5	.29
Empathic attunement (1.130)	Huozi3.5 (0.327)	−0.804 (−0.950 to −0.655)	95.5	<**.**001
Empathic attunement (1.130)	YuanChat-GLM4-9B (1.025)	−0.105 (−0.268 to 0.057)	873.0	.29
Empathic attunement (1.130)	GLM-4-9B-Chat (0.973)	−0.157 (−0.311 to −0.005)	737.5	.28
Empathic attunement (1.130)	CharacterGLM (1.521)	+0.391 (0.234 to 0.548)	497.5	<.001
Empathic attunement (1.130)	GPT-4 (0.709)	−0.421 (−0.611 to −0.229)	522.5	<.001
Supportive strategy (1.311)	YuanChat-Huozi3.5 (1.389)	+0.079 (−0.023 to 0.182)	764.0	.38
Supportive strategy (1.311)	Huozi3.5 (1.070)	−0.241 (−0.363 to −0.120)	533.0	.002
Supportive strategy (1.311)	YuanChat-GLM4-9B (1.252)	−0.059 (−0.161 to 0.043)	729.0	.38
Supportive strategy (1.311)	GLM-4-9B-Chat (0.911)	−0.400 (−0.529 to −0.271)	303.5	<.001
Supportive strategy (1.311)	CharacterGLM (1.436)	+0.125 (0.005 to 0.241)	763.0	.17
Supportive strategy (1.311)	GPT-4 (1.196)	−0.114 (−0.229 to 0.002)	758.0	.18
Perceived helpfulness (1.218)	YuanChat-Huozi3.5 (1.257)	+0.039 (−0.098 to 0.173)	830.5	>.99
Perceived helpfulness (1.218)	Huozi3.5 (1.230)	+0.013 (−0.143 to 0.168)	1080.0	>.99
Perceived helpfulness (1.218)	YuanChat-GLM4-9B (1.173)	−0.045 (−0.188 to 0.102)	860.5	>.99
Perceived helpfulness (1.218)	GLM-4-9B-Chat (0.852)	−0.366 (−0.527 to −0.205)	465.5	<.001
Perceived helpfulness (1.218)	CharacterGLM (1.357)	+0.139 (0.005 to 0.273)	814.0	.21
Perceived helpfulness (1.218)	GPT-4 (1.218)	0.000 (−0.146 to 0.148)	1052.0	>.99
Safety and professional boundaries (1.689)	YuanChat-Huozi3.5 (1.691)	+0.002 (−0.052 to 0.055)	698.0	.87
Safety and professional boundaries (1.689)	Huozi3.5 (1.445)	−0.245 (−0.300 to −0.189)	84.5	<.001
Safety and professional boundaries (1.689)	YuanChat-GLM4-9B (1.598)	−0.091 (−0.138 to −0.045)	380.5	<.001
Safety and professional boundaries (1.689)	GLM-4-9B-Chat (1.427)	−0.263 (−0.316 to −0.209)	42.0	<.001
Safety and professional boundaries (1.689)	CharacterGLM (1.716)	+0.027 (−0.023 to 0.075)	474.5	.32
Safety and professional boundaries (1.689)	GPT-4 (1.523)	−0.166 (−0.216 to −0.116)	133.0	<.001

a The mean ratings were calculated by first averaging the ratings of the 8 experts within each scenario and then averaging these scenario-level means across the 70 scenarios. The 95% CIs were obtained from 100,000 paired bootstrap resamples using the dialogue scenario as the resampling unit.

### Performance of the E-URIAL Framework (RQ2)

To assess inference-time alignment, we compared zero-shot prompting with E-URIAL using 1, 3, or 10 retrieved exemplars. Table 11 presents the results.

**Table 11.** E-URIAL^a^ results.

LLM^b^ and prompt learning	EIS^c^, mean	EES^d^, mean	Human assessment, mean		
			Flu.^e^	Emp.^f^	Help.^g^
GPT-4					
Zero shot	0.45	0.54	0.95	0.76	0.55
E-URIALK=1	0.52	0.58	0.94	0.72	0.63
E-URIALK=3	0.57	0.65	0.95	0.86	0.75
E-URIALK=10	0.57	0.66	0.97	0.85	0.70
ERNIE 4.0					
Zero shot	0.41	0.41	0.91	0.72	0.48
E-URIALK=1	0.44	0.50	0.92	0.77	0.54
E-URIALK=3	0.50	0.53	0.93	0.81	0.57
E-URIALK=10	0.47	0.51	0.93	0.79	0.55
CharacterGLM					
Zero shot	0.65	0.75	0.91	0.85	0.68
E-URIALK=1	0.71	0.81	0.92	0.85	0.73
E-URIALK=3	0.76	0.84	0.91	0.87	0.80
E-URIALK=10	0.73	0.82	0.93	0.83	0.79
YuanChat-Huozi3.5					
Zero shot	0.59	0.68	0.93	0.86	0.62
E-URIALK=1	0.66	0.76	0.92	0.85	0.69
E-URIALK=3	0.69	0.79	0.91	0.89	0.74
E-URIALK=10	0.65	0.74	0.93	0.89	0.70

a E-URIAL: empathy untuned large language models with restyled in-context alignment.

b LLM: large language model.

c EIS: Empathy Identification Score.

d EES: Empathic Expression Score.

e Flu.: fluency.

f Emp.: empathy.

g Help.: Perceived helpfulness.

#### Effect of the Number of Retrieved Exemplars

As Table 11 shows, E-URIAL produced higher scores than zero-shot prompting on most metrics and models. Performance generally improved from k=1 to k=3, whereas k=10 provided little additional improvement. For YuanChat-Huozi3.5, k=3 produced the highest EIS and human-rated empathy scores among the tested settings. GPT-4’s human-rated empathy score decreased slightly from 0.86 at k=3 to 0.85 at k=10. Among the 3 exemplar settings examined, k=3 therefore produced the strongest overall results.

#### Comparison Across Models

Model rankings differed across the evaluation metrics. At k=3, YuanChat-Huozi3.5 achieved a slightly higher human-rated empathy score (0.89 vs 0.87). However, it received lower EIS (0.69 vs 0.76), EES (0.79 vs 0.84), and perceived helpfulness scores (0.74 vs 0.80). Fluency was the same for both models at 0.91.

#### Role of Retrieved Exemplars

E-URIAL selected response examples according to their semantic similarity to the current dialogue context and included them in the model prompt. Scores generally increased from k=1 to k=3 but did not improve consistently when k increased to 10. This pattern shows that the number of exemplars affected performance under the evaluated conditions.

### Ablation Study

To verify the necessity of each E-URIAL component, we conducted an ablation study on YuanChat-Huozi3.5 (k=3). We compared the full model (E-URIAL) against 3 variants: w/o emotion (removing emotion labels), w/o strategy (removing ESC stage and counseling-technique labels), and w/o retrieval (using static, generic exemplars). To isolate the impact of specific variables, all variants retained the standard [label] formatting markers and system instructions. Furthermore, system instruction and random seeds were fixed across all experimental groups to ensure reproducibility of the findings. Table 12 shows the ablation experiment results.

**Table 12.** Ablation results of different components of E-URIAL^a^.

Model variant	EIS^b^	↓^c^	EES^d^	↓	EAC^e^	↓	Emp.^f^	↓
Full model	0.69	—^g^	0.79	—	0.84	—	0.89	—
w/o emotion	0.62	0.07	0.71	0.08	0.77	0.07	0.86	0.03
w/o strategy	0.65	0.04	0.74	0.05	0.79	0.05	0.80	0.09
w/o retrieval	0.60	0.09	0.68	0.11	0.74	0.10	0.73	0.16

a E-URIAL: empathy untuned large language models with restyled in-context alignment.

b EIS: Empathy Identification Score.

c ↓ indicates the decrease relative to the full model.

d EES: Empathic Expression Score.

e EAC: empathy ability calculation.

f Emp.: empathy.

g Not applicable.

Consistent with the evaluation framework, we used a combination of automated evaluation metrics and human assessment. To ensure evaluation efficiency while focusing on the core research objective, we selected empathy (Emp.) as the representative human metric.

Dynamic retrieval was the cornerstone (w/o retrieval). Replacing dynamic retrieval with static exemplars caused the most extreme performance drop across all metrics (eg, EES decreased by 0.11). This indicated that the effectiveness of ICL in emotional support tasks could be considerably influenced by the semantic alignment between the exemplars and the current query.

Explicit labels improved response generation. Removing emotion labels reduced automated evaluation performance (eg, EES decreased by 0.08) and weakened the model’s ability to track changes in the seeker’s emotional state. Removing ESC and counseling technique labels resulted in more generic expressions of comfort and reduced empathy ratings by 0.09. Qualitative analysis showed that these models frequently produced general responses such as “I’m sorry to hear that” instead of applying specific counseling techniques such as clarification or support. The results also supported the synergistic effect reported in previous research [22]: emotion labels represented the seeker’s emotional state, ESC and technique labels guided response strategy, and retrieved exemplars influenced response style.

### Qualitative Analysis

To concretely illustrate the impact of dynamic retrieval, we analyzed a representative case from our test set. The removal of retrieval mechanisms resulted in a loss of strategic depth (Table 13).

**Table 13.** Case study: impact of dynamic retrieval; user query: “yes, he brought it up, and we had a huge fight.”

Model variant	Generated response	Analysis
w/o retrieval (static exemplars)	“Fighting is indeed unpleasant. Perhaps you can try to communicate well.”	Generic comfort: Without context-specific exemplars, the model reverts to a “safe” baseline. It recognizes the negative sentiment (“unpleasant”) but offers a passive suggestion for communication.
Full model (E-URIAL^a^)	“It is indeed sad and infuriating. [Validation] When you argued, what point did you most want him to understand? [Clarification]”	Strategic support: The model (1) accurately labels the emotion mixture (sadness and anger) and (2) executes a specific clarification strategy to pivot the conflict into a constructive exploration of needs.

a E-URIAL: empathy untuned large language models with restyled in-context alignment.

Taken together, the ablation results indicated that dynamic retrieval was the most influential component. Emotion and strategy labels collectively served as indispensable guide rails that reduced empathy drift and superficial responding. These findings suggested that each component of E-URIAL contributes uniquely to empathic response quality and that the framework performs optimally when all 3 elements operate in concert.

### Error Analysis

While the E-URIAL framework enhanced empathic performance, a qualitative analysis of outlier cases suggested 2 primary failure modes. First, contextual contamination, or exemplar drift, explained the diminishing returns observed when k> 3. It occurred when retrieved exemplars, namely language stylistic anchors, introduced linguistic cues from mismatched life contexts (eg, using workplace terminology to address academic stress). Even if the predicted strategy labels were correct, these “leaked” cues could dilute the response’s relevance, suggesting that semantic precision in retrieval was more important than mere exemplar quantity. Second, strategic misalignment occurred when the model overrelied on problem-solving, where the model bypassed the [Comfort] stage and moved prematurely to [Action].

## Discussion

Experimental results suggested that the integration of strategic SFT (YuanChat) and dynamic inference-time alignment (E-URIAL) created a robust framework for professional emotional support.

### Synergy Between Fine-Tuning and Retrieval

YuanChat-Huozi3.5 achieved its strongest overall performance when combined with E-URIAL, suggesting that SFT and inference-time guidance played complementary roles. Fine-tuning on ECD-3 improved empathy-related performance relative to the backbone model. E-URIAL provided additional context-specific guidance through predicted emotion and strategy labels and dynamically retrieved counselor exemplars, without further parameter updates. The ablation results showed the largest performance decline when dynamically retrieved exemplars were replaced with static exemplars, indicating that contextually relevant demonstrations were an important contributor under the evaluated conditions. Together, these findings suggest that combining domain-specific parameter adaptation with retrieval-based guidance could improve the contextual appropriateness and strategic consistency of emotional support responses.

### Parameter Efficiency and Domain Specialization

The performance gains of YuanChat-GLM4-9B over its GLM-4-9B-Chat backbone suggest that a relatively compact model can benefit from domain-specific, structured supervision. Fine-tuning on ECD-3 improved its empathy identification, empathic expression, and human-rated empathy across both evaluation datasets. These results indicate that parameter-efficient adaptation using counseling-informed data could enable relatively compact models to achieve competitive performance on empathy-related tasks.

### Effect of Exemplar Quantity

Across the evaluated models and metrics, k=3 generally provided the strongest overall performance, whereas increasing the number of retrieved exemplars to k=10 yielded limited additional gains and, in several cases, lower scores. This pattern suggests diminishing returns as the number of exemplars increased under the evaluated conditions.

At k=3, a small set of highly ranked exemplars may have provided sufficient contextual and stylistic guidance for generating responses consistent with the predicted emotion and strategy labels. Increasing k to 10 may have introduced redundant or less relevant contextual information, thereby increasing prompt complexity and reducing the relative prominence of the current dialogue and its associated labels. Prompt length represents another possible explanation. As the model input was limited to 4096 tokens, longer prompts may have increased the likelihood of truncation or reduced the effective influence of relevant information.

### Theoretical Contributions

This study makes 3 theoretical contributions. First, it conceptualizes machine empathy as the coordination of strategic intent and linguistic realization across changing dialogue contexts. Strategic intent concerns the selection of an appropriate emotional support stage and counseling technique, whereas linguistic realization concerns how that strategy is expressed through empathic language. This distinction connects counseling theory with computational response generation and provides an interpretable account of how LLMs can adapt their responses to users’ emotional and support needs.

Second, the findings clarify the role of explicit, theory-informed labels in empathic response generation. The ablation results showed that removing emotion or strategy labels reduced performance, suggesting that these labels help models identify the user’s emotional state, select an appropriate supportive action, and express that action in language. The results support the use of structured intermediate representations in domain-specific fine-tuning and inference-time guidance. They also suggest that structured domain knowledge can help smaller models achieve competitive performance, although the present experiments did not directly compare different levels of label granularity.

Third, the findings provide evidence about how dynamic retrieval contributes to inference-time alignment. Replacing dynamically retrieved exemplars with static exemplars produced the largest performance decline in the ablation study. Performance generally improved when the number of exemplars increased from 1 to 3 but did not improve consistently with 10 exemplars. These results indicate that contextually relevant demonstrations can improve strategy realization, while additional exemplars may introduce less relevant information. The effectiveness of retrieval-augmented ICL therefore depends on both exemplar relevance and exemplar quantity.

### Practical Implications

The YuanChat models and E-URIAL framework have several practical implications for emotional support systems. First, the performance of YuanChat-GLM4-9B suggests that relatively compact models can benefit from domain-specific fine-tuning and structured inference-time guidance. Second, the construction of ECD-3 provides a practical approach to converting real-world Chinese counseling dialogues into structured training data while incorporating counseling theory, label-consistency filtering, and privacy safeguards. This approach may inform the development of similar datasets in other languages and cultural contexts. Third, the distinction between strategic intent and linguistic realization may be applicable to other forms of supportive communication, including health care communication and personalized education. Further domain-specific validation would be required before applying the framework in these settings.

### Limitations and Future Directions

First, although the ECD-3 dataset provided high-quality counseling dialogues, its source from a single Chinese company could introduce sample homogeneity; therefore, the current findings are primarily grounded in Chinese emotional support settings. Future researchers should incorporate cross-cultural and multisource mental health data to enhance generalizability. Second, although differentiated PEFT methods and the auxiliary use of GPT-4 for label filtering were controlled for consistency, future studies would require the employment of cross-validation across diverse architectures and broader baseline models to further mitigate potential methodological biases. Third, the proposed framework was evaluated in emotional support contexts; its translation into formal clinical mental health settings would require meeting substantially more rigorous standards, including expert clinical oversight, systematic safety evaluation, risk management protocols, and validation through real-world clinical trials. Future work will therefore focus on strengthening the clinical applicability and real-world deployment. Fourth, acknowledging the inherent variability in LLM outputs for complex emotional tasks, we plan to implement multirun stability quantification using metrics such as text consistency coefficients to ensure relatively robust performance evaluations in professional psychological scenarios.

Finally, future work should address the potential confusion between empathy and sycophancy. Although both empathy and sycophancy may involve emotionally responsive language, they differ in function. Empathy is oriented toward long-term psychological well-being and, when necessary, challenges users’ maladaptive cognitions. Sycophancy is driven by users’ immediate satisfaction; it uncritically agrees with users and avoids conflict [76]. The evaluation framework in this study already contains implicit safeguards against sycophancy. For example, the challenge category of counseling techniques includes identifying contradictions in users’ narratives and questioning maladaptive beliefs, and the negative linguistic cues in the EES metric, such as fabricated conclusions and uncritical agreement, can penalize overly accommodating responses. However, this study did not include an explicit sycophancy detection metric. Future research should incorporate explicit detection mechanisms into the evaluation framework and use long-term satisfaction rather than immediate satisfaction to assess empathic quality.

### Conclusions

This study highlighted 3 contributions that advanced emotional support systems. First, it helped narrow the gap between generic consolation and professionally grounded emotional support by training on the ECD-3 dataset, which provided a foundational resource for emotional support research and model development. Second, it optimized empathic alignment via the E-URIAL method, which operationalized composite strategy, strategic intent, and linguistic realization to improve stylistic coherence. Third, the findings suggest that structured, fine-grained strategy labels may help improve the emotional support performance of smaller models.

By embedding professional counseling techniques into AI-driven dialogue, our findings pave the way for scalable and strategy-aware digital interventions that can effectively complement traditional psychotherapy and expand the accessibility of preliminary mental health support. The findings also suggest the potential value of such systems for medical mental health settings, particularly in expanding access to scalable and context-sensitive supportive interactions. At the same time, their contribution to health care should be understood as clinically promising rather than clinically established, given the continued need for stronger external validation, standardized outcome assessment, and explicit safety governance.

Building on these findings, several directions remain for future exploration. One promising avenue is cross-cultural empathy adaptation, which would involve expanding the dataset and framework to incorporate diverse cultural and linguistic nuances, thereby enhancing generalizability and robustness. Another direction concerns stability and reliability quantification, focused on developing methods to measure and control output variability in emotionally sensitive interactions, with the aim of obtaining consistent emotional support quality across different runs. A third direction is multimodal emotional perception, which would extend the E-URIAL framework to integrate nonverbal cues such as voice and facial expressions, enabling a more comprehensive human-machine empathic interaction.

## Supplementary material

10.2196/95603 Multimedia Appendix 1 Empathy computing model.

10.2196/95603 Multimedia Appendix 2 Expert validation protocol.

10.2196/95603 Multimedia Appendix 3 Situational reasoning item example.

10.2196/95603 Multimedia Appendix 4 Input formatting.

10.2196/95603 Multimedia Appendix 5 Adapter hyperparameters.

10.2196/95603 Multimedia Appendix 6 Empathy effect evaluation model.

10.2196/95603 Multimedia Appendix 7 Validation of the EPITOME evaluator in Chinese context.

10.2196/95603 Multimedia Appendix 8 25 Linguistic cues.

10.2196/95603 Multimedia Appendix 9 ECD-3 dataset (partial release): annotated counselor-response turns in JSON format.

10.2196/95603 Checklist 1 TRIPOD+LLM checklist.

## Abbreviations

BLEU: Bilingual Evaluation Understudy
DoRA: weight-decomposed low-rank adaptation
E-URIAL: empathy untuned large language models with restyled in-context alignment
EAC: empathy ability calculation
EES: empathic expression score
EIS: empathy identification score
ER: emotional reaction
ESC: emotional support conversation
EX: explorations
GPU: graphics processing unit
ICL: in-context learning
IP: interpretations
IRR: interrater reliability
LLM: large language model
PEFT: parameter-efficient fine-tuning
QDoRA: quantized weight-decomposed low-rank adaptation
RQ: research question
SBERT: sentence bidirectional encoder representations from transformers
SFT: supervised fine-tuning
TRIPOD+LLM: Transparent Reporting of a Multivariable Prediction Model for Individual Prognosis or Diagnosis–Large Language Model
URIAL: untuned large language models with restyled in-context alignment
