# Supplementary material for: A Multistage Fine-Tuning and Empathy Alignment Approach for an Emotional Support Dialogue System: Development and Evaluation Study
Source: J Med Internet Res. 2026 Oct 29;28:e95603. doi: 10.2196/95603 (PMC13719010; doi:10.2196/95603)
Supplement: Supplementary file 2 [file jmir-v28-e95603-s002.docx]

**Multimedia Appendix 5: Expert Validation Protocol**

**A.1 Evaluation Unit and Sampling**

Evaluation Unit: The counselor-response turn, defined as “a single counselor response alongside its preceding dialogue context.”

Sampling Method: Stratified random sampling was applied to the synthetic dataset (D_syn). The sampling was stratified by "topic × ESC stage” to ensure comprehensive coverage of primary topics and label categories.

Sample Size: N = 216 counselor-response turns.

Evaluators: Two professionals, scoring independently.

**A.2 Scoring Dimensions and Behavioral Anchors (5-Point Scale)**

**Dimension 1: Situational Realism**

1 Point: Clearly inconsistent with a real counseling context; deviates from context comprehension or provides irrelevant responses; tone resembles templated preaching or "chicken soup"; fails to capture key emotions and core issues.

2 Points: Intermediate between 1 and 3.

3 Points: Generally aligns with the counseling context and capable of responding to main emotions or issues, but somewhat generalized; average level of individualization, lacking context-specific follow-up questions or clarification.

4 Points: Intermediate between 3 and 5.

5 Points: Highly tailored to the context and the client's narrative; accurately captures key emotional cues and core issues; the response structure aligns with the logical flow of counseling interactions (e.g., exploration—comfort—support/guidance) and naturally builds upon the previous dialogue turn.

**Dimension 2: Empathic Appropriateness**

1 Point: Empathy is absent or inappropriate (e.g., denial, judgment, or belittling); explicitly incorrect emotional reflection; hollow comfort or over-assurance; likely to trigger client defensiveness.

2 Points: Intermediate between 1 and 3.

3 Points: Expresses basic understanding and support (emotional reflection/validation), but lacks depth; insufficient clarification of the client's needs and feelings; the response appears somewhat "standardized."

4 Points: Intermediate between 3 and 5.

5 Points: Empathy is precise and well-measured; accurate emotional reflection and adequate meaning clarification; supportive without overstepping professional boundaries or making decisions for the client; promotes a sense of safety and encourages further expression.

**Dimension 3: Semantic Fidelity and Naturalness**

1 Point: The response is clearly inconsistent with the preceding context or original meaning; obvious "fabrication or addition of key facts/advice"; sentences are stiff, resembling spliced templates or machine translation, reading unnaturally.

2 Points: Intermediate between 1 and 3.

3 Points: Overall semantics are largely consistent with no obvious addition of key facts, though minor local deviations or over-generalization may exist; language is generally fluent but still shows signs of being templated, lacking a colloquial feel, or not fully reflecting a genuine counseling tone.

4 Points: Intermediate between 3 and 5.

5 Points: Completely maintains consistency with the original meaning and key details without adding unverified facts or unnecessary inferences; language is highly natural, closely matching real counseling contexts and tones; readers can barely distinguish it as an AI-generated or rewritten response.

**A.3 Evaluation Record Sheet (Expert Item-by-Item Template)**

Sample ID: ____________

Situational Realism (1–5): ____ Remarks: ________________________

Empathic Appropriateness (1–5): ____ Remarks: ________________________

Semantic Fidelity & Naturalness (1–5): ____ Remarks: ________________________
