# Supplementary material for: A Multistage Fine-Tuning and Empathy Alignment Approach for an Emotional Support Dialogue System: Development and Evaluation Study
Source: J Med Internet Res. 2026 Oct 29;28:e95603. doi: 10.2196/95603 (PMC13719010; doi:10.2196/95603)
Supplement: Supplementary file 3 [file jmir-v28-e95603-s003.docx]

**Multimedia Appendix 4: Situational Reasoning Item Example**

Scenario:

Xiaozhou made a mistake during an important presentation. Afterward, a classmate reassured her, saying, “It’s okay; no one will think much of it.” She smiled and replied, “I’m fine,” but then declined an invitation to have dinner with her classmates. After returning to her dormitory, she repeatedly checked the brief message she had received from her academic advisor and revised the presentation materials several times, even though they had already been submitted. When a friend later messaged her, “You already did a great job,” she replied only, “Mm, I’d like to be alone for a while.”

Question:

Which option most accurately describes Xiaozhou’s likely thoughts, feelings, and behavioral intentions?

A. She is no longer concerned about the mistake and simply wants to rest because she is tired.

B. She may feel embarrassed and anxious, worry about how others perceive her, and intend to withdraw temporarily while regaining a sense of control by repeatedly reviewing her work.

C. She is angry about her classmates’ attempts to reassure her and intends to end her relationships with them.

D. She has little emotional reaction to the presentation and is revising the materials only as part of her usual study habits.

E. She believes that she performed better than her classmates and wants to demonstrate her competence by spending time alone.

Intended Answer: B

Expert Rationale (Not Presented to the Evaluated Models):

Option B integrates both the verbal and behavioral cues in the scenario: the discrepancy between Xiaozhou’s statement that she is “fine” and her subsequent social withdrawal, her repeated checking of her advisor’s message, and her continued revision of materials that had already been submitted. Together, these cues suggest embarrassment or anxiety, concern about negative evaluation, and an intention to withdraw temporarily and regain a sense of control through repeated checking.

Constructs Assessed:

- Emotion inference: embarrassment and anxiety

- Thought inference: concern about being negatively evaluated

- Intention inference: temporary social withdrawal and an attempt to regain control

During the formal evaluation, only the scenario, question, and response options were presented to the models. The intended answer and expert rationale were withheld.
