# Supplementary material for: A Multistage Fine-Tuning and Empathy Alignment Approach for an Emotional Support Dialogue System: Development and Evaluation Study
Source: J Med Internet Res. 2026 Oct 29;28:e95603. doi: 10.2196/95603 (PMC13719010; doi:10.2196/95603)
Supplement: Supplementary file 4 [file jmir-v28-e95603-s004.docx]

**Multimedia Appendix 6: Input Formatting**

For YuanChat-GLM4-9B (GLM-4-9B-Chat backbone), the input was “[gMASK] sop <|system|>{*system prompt*}<user|>*{Seeker prompt 1}*<|assistant|>*{assistant response 1}...*<|user|>*{Seeker prompt N}*<|assistant|>*{assistant response N}*<eos>.” For YuanChat-Huozi3.5 (Huozi3.5 backbone), we used the corresponding instruction format: is “<s>[INST] {*Seeker prompt 1}* [/INST] {*assistant response 1}* </s>...[INST] {*Seeker prompt N}* [/INST] {*assistant response N}* </s>”.
