# Supplementary material for: A Multistage Fine-Tuning and Empathy Alignment Approach for an Emotional Support Dialogue System: Development and Evaluation Study
Source: J Med Internet Res. 2026 Oct 29;28:e95603. doi: 10.2196/95603 (PMC13719010; doi:10.2196/95603)
Supplement: Supplementary file 5 [file jmir-v28-e95603-s005.docx]

**Multimedia Appendix 7: Adapter hyperparameters**

Table 8 summarizes the core adapter hyperparameters used for parameter-efficient finetuning across the two backbone models, including their search spaces, selected values, and the model-specific rationale behind each choice.

**Table 8. Adapter Core Parameters (Model-Specific)**

| **Hyperparameter** | **Symbol** | **Search Space** | **GLM-4-9B (DoRA)** | **Huozi3.5 (QDoRA)** | **Description** |
| --- | --- | --- | --- | --- | --- |
| Rank | r | {8, 16, 32, 64} | 64 | 32 | Sparse MoE models converge with lower ranks |
| Scaling Factor | α | {16, 32, 64, 128} | 128 (2r) | 64 (2r) | Controls adapter update magnitude |
| Dropout | p | {0.0, 0.05, 0.1} | 0.05 | 0.1 | Higher dropout for quantized models to prevent overfitting |
| Quantization Type | q | {fp16, nf4} | fp16 | nf4 | Huozi3.5 requires 4-bit NF4 quantization |
| Double Quantization | - | {True, False} | FALSE | TRUE | Enabled only for QDoRA to reduce memory footprint |
| Target Modules | - | - | All linear layers | Active experts only | Avoid finetuning inactive expert parameters |
