# Supplementary material for: A Multistage Fine-Tuning and Empathy Alignment Approach for an Emotional Support Dialogue System: Development and Evaluation Study
Source: J Med Internet Res. 2026 Oct 29;28:e95603. doi: 10.2196/95603 (PMC13719010; doi:10.2196/95603)
Supplement: Supplementary file 6 [file jmir-v28-e95603-s006.docx]

**Multimedia Appendix 2: Empathy Effect Evaluation Model**

***A. Model basis and workflow***

Our empathy computation model is a utility model for assessing the empathy quality of LLM-generated replies. It infers empathy-related linguistic cues from a dialogue and aggregates them into an empathy score. We use ChatGLM3-6B as the backbone because it offers strong Chinese NLI performance, a moderate size (<10B) suitable for private deployment, and a higher normalized empathy-evaluation score (0.672) than comparable open-source baselines (e.g., LLaMA2-7B: 0.481, Baichuan2-7B-Base: 0.588).

*Three-step workflow.*

1. Input formatting. We take a dialogue turn (“Seeker” + “Assistant”) and serialize it into Instruction + Input, where the instruction states the task “evaluate empathy cues in the assistant reply.”
2. Cue inference. The model predicts a set of 25 reduced empathy cues learned in training (15 positive, e.g., “on-topic, concrete response,” “validate seeker’s feelings”; 10 negative, e.g., “unsupported conclusions,” “deny the seeker”).
3. Scoring & explanation. We count positive/negative hits with predefined weights to produce an overall empathy score, and we list contributing cues to keep the evaluation interpretable.

***B. Data and training (methods)***

*From cue design to training data*

- Cue reduction. Starting from human-derived empathy cues, we removed cues infeasible for LLMs (e.g., “interrupting the speaker”), rewrote the remaining cues into promptable forms, and finalized 25 cues (14 positive, 11 negative) as the evaluation basis
- Training set construction (ChatGPT-4 + Self-Instruct).
  • Augmentation method. For each cue, we designed one dialogue example using ICL + CoT across 11 everyday scenarios (social difficulty, relationships, workplace stress, etc.), prompting ChatGPT-4 to generate cue-conforming and non-conforming dialogues.
  • Triplet format. (Instruction, Input, Output[a1 + a2]), where a1 lists true cues hit by the reply and a2 lists distractor (false) cues.
  • Scale & quality. ~11k training instances covering 11 scenarios; we cleaned low-quality items so the model learns to accept correct cues and reject wrong ones.
- Test set (human-audited).
  We sampled dialogues from ECD-3, obtained independent cue predictions from ChatGPT-4 / ERNIE-4.0 / GLM-4, and consolidated them. Three trained raters adjudicated disagreements and removed items with <3 cues. Inter-annotator agreement Cohen’s κ > 0.85; final size 100.

***C. Training setup***

- Environment & framework. 4× NVIDIA A100-SXM4-80GB; DeepSpeed ZeRO-2 + LoRA; FP16 loading.
- Key hyperparameters: AdamW, LR 3e-5, 3 epochs, max_source_length=2048, max_target_length=2048, per-GPU batch 4, save_steps=1000.
- Objective. Token-level cross-entropy; the model learns to map dialogues to correct cues by distinguishing a1 (true) from a2 (false) cues.

***D. Strategy prediction from cues***

We map inferred cues → empathy strategies:

Cue–strategy links. Example:

- *Affective validation* ⇢ positive cues Y3 (speak from seeker’s perspective), Y11 (validate feelings), Y12 (accurate reflection).
- *Guided exploration* ⇢ Y5 (Socratic questions), Y9 (guide problem finding).
- *Avoid negation* ⇢ absence of negative cues N4 (directly deny seeker), N10 (ignore contradictions).
- Decision rule. If ≥2 positive cues for a strategy are hit and no conflicting negative cue is hit, we mark that strategy. If multiple strategies qualify, we rank by the number of supporting cues and report the dominant one.
- Output. The prediction includes the selected strategy and the supporting cues (e.g., “Affective validation; supported by Y11, Y12”).

***E.Evaluation and comparison***

Setup. We evaluate our empathy computing model and 13 mainstream LLMs (7 closed-source: GPT-4, ERNIE-4.0, …; 6 open-source: Qwen1.5-72B-Chat, ChatGLM3-6B, …) on cue prediction (hit the 25 cues). Metrics: Accuracy and F1. Test set: 100 human-audited items (mean IAA 0.86).

**Results**

| LLM | Open source | acc. | f1 |
| --- | --- | --- | --- |
| **GPT4** | No | **.9221** | **.9346** |
| ERNIE 4.0 | No | .8912 | .8623 |
| **GLM4** | No | **.9372** | **.9138** |
| Qwen2.1 | No | .904 | .9103 |
| ERNIE 3.5 | No | .8736 | .868 |
| GLM3 | No | .8823 | .8542 |
| Qwen1.5-72B-Chat | Yes | .8593 | .8679 |
| GPT3.5 | No | .8605 | .8303 |
| ChatGLM3-6B | Yes | .826 | .8129 |
| Qwen1.5-14B-Chat | Yes | .8018 | .7967 |
| Baichuan2-7B-Chat | Yes | .7634 | .783 |
| deepseek-llm-67b-chat | Yes | .7347 | .7207 |
| internlm2-chat-20b | Yes | .7206 | .7113 |
| Empathy computing model | / | **.8456** | **.8253** |

Observations*.*

1.Closed-source models lead overall (GLM-4/GPT-4).

2.Our empathy computing model, despite its small size, is competitive among open-source models and approaches some closed-source baselines (e.g., Acc. ≈ 90.2% of GLM-4; F1 ≈ 88.3% of GPT-4).

3.Compared with its backbone ChatGLM3-6B, empathy computing model improves Acc. by +2.37% and F1 by +1.52%, supporting the effectiveness of our training design.

***F. Interpretability***

Our empathy computing model is designed to be explainable end-to-end:

1.Transparent basis. Scoring relies on 25 clearly defined cues (e.g., Y1 “stay on topic; address the current issue,” N2 “blame or scold the seeker”), avoiding “black-box” judgments.

2.Traceable reasoning. For each cue, the model outputs YES/NO plus short rationale (see Table 4.18 examples), so readers can verify whether the judgment matches the dialogue.

3.Attribution view. The final empathy score is a weighted sum of cue hits; we show which positive cues raised the score and which negative cues lowered it (e.g., “Score 80; driven by Y3 +15, Y11 +12; no negative hits”).

Finally, alignment with human judgments is supported by the human-audited test set (κ > 0.85), which provides an external check on the model’s cue-level decisions.
