# Supplementary material for: A Multistage Fine-Tuning and Empathy Alignment Approach for an Emotional Support Dialogue System: Development and Evaluation Study
Source: J Med Internet Res. 2026 Oct 29;28:e95603. doi: 10.2196/95603 (PMC13719010; doi:10.2196/95603)
Supplement: Supplementary file 7 [file jmir-v28-e95603-s007.docx]

**Multimedia Appendix 8: Validation of the EPITOME Evaluator in Chinese Context**

The Chinese version retained the three dimensions and three-level rating scheme of the original EPITOME framework. Emotional Reactions refer to expressions of concern, warmth, or compassion in response to the seeker’s experiences. Interpretations refer to contextually relevant expressions of understanding regarding the emotions and experiences explicitly or implicitly communicated by the seeker. Explorations refer to contextually relevant questions intended to elicit emotions or experiences that have not yet been fully expressed. Each dimension was rated as no expression (0), weak expression (1), or strong expression (2). Strong expressions required an explicit connection to specific emotions, experiences, or circumstances described by the seeker. General expressions of comfort, declarations of understanding, and generic questions were typically rated as weak.

We selected 8,342 seeker–response pairs from Dgold. The data were divided into training, validation, and test sets containing 5,840, 1,251, and 1,251 pairs, respectively. Dgold cases used for the automatic evaluation of YuanChat were not included in the training or validation sets of the evaluator.

We recruited 10 master’s students in clinical psychology through an open call at the authors’ institution. Before formal annotation, all annotators received standardized training on the operational definitions of the three EPITOME dimensions, the boundaries between the 0–2 rating levels, typical expressions in Chinese-language contexts, and potentially ambiguous cases.

To adapt EPITOME to Chinese-language contexts, we adopted the bi-encoder architecture used for its empathy identification task, replaced the original encoder with Chinese RoBERTa-wwm-ext, and used the annotated Chinese data described above to train and internally validate the evaluator [1].

Each input comprised a seeker text and its corresponding response. We used a seeker encoder (S-Encoder) and a response encoder (R-Encoder) to encode the two texts separately. Both encoders were initialized with Chinese RoBERTa-wwm-ext but were updated independently during fine-tuning without parameter sharing. A single-head attention mechanism was then used to integrate the representation of the seeker text into the response representation, producing a context-aware representation for predicting the expression level of each empathy mechanism. Following the original EPITOME modeling approach, we trained three structurally identical but parametrically independent three-class models for Emotional Reactions (ER), Interpretations (IP), and Explorations (EX). Each model predicted an expression level of 0, 1, or 2, corresponding to no, weak, or strong expression, respectively. The maximum input length was 256 tokens for both seeker and response texts. The three models were trained separately using the AdamW optimizer, a learning rate of $2\times10^{-5}$，a weight decay of 0.01, an effective batch size of 32, and a maximum of 4 epochs. The random seed was set to 42. Each model was optimized using three-class cross-entropy loss. Macro-F1 was calculated on the validation set after each epoch, and the checkpoint with the highest validation macro-F1 was retained separately for ER, IP, and EX. Given the maximum training duration of 4 epochs, no additional early-stopping criterion was applied.

On the test set, the Chinese EPITOME evaluator achieved an accuracy/macro-F1 of 0.8875/0.7457 for ER, 0.8599/0.7468 for IP, and 0.8850/0.7189 for EX.

For response $i$，we summed the predicted expression levels for the three dimensions and divided the sum by the maximum possible score of 6 to obtain a normalized response-level EIS:

$$EIS_{i}=\frac{ER_{i}+IP_{i}+EX_{i}}{6}$$

Where $ER_{i}、IP_{i}和EX_{i}\in\{0,1,2{\}}_{.}$ For an evaluated model with N responses, the overall EIS was defined as:

$$EIS=\frac{1}{N}\sum_{i=1}^{N} EIS_{i}$$

EIS ranges from 0 to 1, with higher values indicating stronger overall expression of the three empathy mechanisms identified by the evaluator.

[1] Cui Y, Che W, Liu T, et al. Pre-training with whole word masking for Chinese BERT. IEEE/ACM Trans Audio Speech Lang Process. 2021;29:3504-3514. [doi: 10.1109/TASLP.2021.3124365]
