# Supplementary material for: A Multistage Fine-Tuning and Empathy Alignment Approach for an Emotional Support Dialogue System: Development and Evaluation Study
Source: J Med Internet Res. 2026 Oct 29;28:e95603. doi: 10.2196/95603 (PMC13719010; doi:10.2196/95603)
Supplement: Supplementary file 8 [file jmir-v28-e95603-s008.docx]

**Multimedia Appendix 3: 25 Linguistic Cues**

The above linguistic cues are derived from the D_gold. Cues that only appear in human-to-human conversations (e.g., "interrupting the visitor midway") are removed, and the remaining cues are rewritten into verifiable prompts. Finally, 14 positive cues and 11 negative cues are obtained

Empathetic Linguistic Cues for Prompt Styles

| **ID** | **Positive cues** | **ID** | **Negative cues** |
| --- | --- | --- | --- |
| Y1 | The response sticks to the point and stays focused on the current topic. | N1 | The response includes unsupported or fabricated conclusions or opinions. |
| Y2 | The response is concise and easy to understand. | N2 | The response contains accusatory language or blames the Seeker. |
| Y3 | The response expresses emotions and feelings from the Seeker's perspective. | N3 | The response uses a rhetorical question, such as “Don’t you think so?” |
| Y4 | The response uses first-person plural or third-person phrasing. | N4 | The response directly negates the Seeker’s ideas. |
| Y5 | The response asks questions in an enlightening or choosing manner. | N5 | The response did not objectively assess the questioner’s viewpoint but instead displayed unconditional agreement. |
| Y6 | The response restates or rephrases the Seeker’s feelings or issues in a more specific or understandable way. | N6 | The response is unrelated or irrelevant to the current discussion topic. |
| Y7 | The response asks the Seeker, using phrasing like “Is that right?” or “Is that how it is?” | N7 | The response is overly verbose, providing excessive information and not focusing on the current topic. |
| Y8 | The response identifies and explains any contradictions between the Seeker's current and previous statements. | N8 | The response contradicts the Seeker’s stated facts, opinions, or thoughts. |
| Y9 | The response uses a guiding method to help the Seeker identify the issue. | N9 | The response asks more than two questions to the Seeker. |
| Y10 | The response gently negates the Seeker's idea. | N10 | The response fails to point out contradictions with the Seeker's previous statements. |
| Y11 | The response affirms the Seeker’s feelings and experiences. | N11 | The response uses first-person singular phrasing. |
| Y12 | The response accurately understands and reflects the Seeker's feelings. |  |  |
| Y13 | The response uses a questioning tone when asking for the Seeker's opinion, like “Is that okay?” |  |  |
| Y14 | The response involves self-disclosure, sharing personal experiences. |  |  |
