# Supplementary material for: A Multistage Fine-Tuning and Empathy Alignment Approach for an Emotional Support Dialogue System: Development and Evaluation Study
Source: J Med Internet Res. 2026 Oct 29;28:e95603. doi: 10.2196/95603 (PMC13719010; doi:10.2196/95603)
Supplement: Supplementary file 10 [file jmir-v28-e95603-s001.doc]

**Multimedia Appendix 1 1: Empathy Computing Model**

***A. Research Objective and Task Definition***

In this study, we model empathy effectiveness as a multi-task emotion recognition problem on counseling dialogues. Our goal is to automatically quantify the empathy quality of counselor responses using text only. The model is used to assist human annotation and analysis; it is not designed for online response generation.

Following the ERC (Emotion Recognition in Conversation) paradigm, each *Seeker question–Counselor reply* pair is treated as one sample. For each reply, the model predicts:

- its ESC stage (Explore / Comfort / Action);
- the counseling technique used (Clarification / Challenge / Support);
- the intensity of the emotional reaction (discrete level);
- the strength of three empathy dimensions: emotional empathy, cognitive empathy, intention understanding;
- an overall Empathy Effectiveness in Counseling score, computed from the above.

***B. Data and Annotation***

Building on the ECD-3 dataset, we construct an extended dataset, referred to as ECD-2. The data still come from real Chinese text-based counseling sessions and are labeled at the turn level by trained annotators. Compared with ECD-3, ECD-2 adds two new annotation fields:

- **Empathy dimensions:** emotional empathy, cognitive empathy, intention understanding, each scored with Level ∈ {0, 1, 2};
- **Emotional reaction:** describing the valence and strength of the counselor’s emotional response, with scores in {-1, 0, 1, 2}.

We also keep the original labels for ESC stage, counseling technique, and overall empathy effectiveness.

Theoretically, we link the three ESC stages to the three empathy dimensions as follows:

Explore: relies on both emotional empathy and cognitive processing;

Comfort: emphasizes emotional empathy and downplays intention analysis;

Action: emphasizes cognitive processing and intention understanding.

Based on the distribution of high- and low-empathy samples, we observe clear dimension preferences in high-empathy replies at different stages, whereas low-empathy replies show a more even, less distinctive distribution across dimensions. This empirical pattern motivates the design of our computational framework.

***C. Empathy Computing Model***

At the theoretical level, we define empathy effectiveness as a combination of emotional reaction × empathy dimensions × stage-specific weights.

For each reply, we first obtain **its emotional reaction score,** and the **scores of the three empathy dimensions**. Then, depending on the reply’s ESC stage, we assign different weights to each empathy dimension (for example, in the Comfort stage we increase the weight of emotional empathy and reduce or suppress the weight of intention understanding). Finally, we aggregate the weighted components into a single scalar, which we use as the empathy effectiveness score for that reply. The formula is

Intuitively, we assume that *“the right type of empathy in the right stage”* constitutes high-quality empathy:

If a Comfort-stage reply focuses too much on giving reasons or advice (intention understanding dominates), it is treated as a stage–dimension mismatch and penalized in the overall score.

If an Explore-stage reply only offers emotional acknowledgment without necessary cognitive clarification, its score is also reduced.

Stage weights are estimated from the empirical distribution of high-empathy samples. The settings are summarized below:

Weight Settings of

|  | **Explore** | **Comfort** | **Action** |
| --- | --- | --- | --- |
| **Emotional empathy** | 0.3 | 0.6 | 0.1 |
| **Cognitive empathy** | 0.2 | 0.2 | 0.6 |
| **Intention understanding** | 0.2 | 0 | 0.8 |

***D. Model Architecture and Training Procedure***

We design a two-stage **empathy computing model,** based on Chinese RoBERTa-wwm-ext. The framework consists of two sub-models:

Sub-model 1: Text Classification

- Tasks: predict ESC stage, counseling technique, and emotional reaction intensity;
- Architecture: a single-encoder RoBERTa classifier, where the current reply is concatenated with a fixed-size window of previous dialogue turns;
- Role: provides automatic labels for stage and emotional reaction, which serve as inputs to the EEC calculation.

Sub-model 2: Empathy Dimension Recognition and Cue Extraction

- Tasks:

score each of the three empathy dimensions (0 / 1 / 2);

highlight words or phrases in the reply that function as empathy cues (language evidence for the predicted dimensions).

- Architecture: a dual-encoder, multi-task model built on RoBERTa-wwm-ext:

S-Encoder encodes dialogue history + current Seeker utterance;

R-Encoder encodes the current reply;

an attention mechanism aligns Seeker-side context with the reply representation.

- Pretraining: both encoders are first further pre-trained on PsyQA and related Chinese mental-health QA corpora for domain adaptation. They are then jointly fine-tuned on ECD-2 in a multi-task setting.

Both sub-models are trained with cross-entropy losses. The overall multi-task loss is obtained by a simple weighted sum of the classification loss and the cue-extraction loss.

***E. Baselines and Main Results (Brief)***

To evaluate the effectiveness of the empathy computing model, we compare it with several widely used baselines.

- Text classification tasks (ESC stage, counseling technique, emotional reaction, empathy dimension prediction)：

**Baselines:** TF–IDF + logistic regression, BERT-base, RoBERTa-base;

**Metrics:** accuracy (acc.) and F1.

**Result:** the proposed empathy computing model outperforms all baselines across tasks and yields on average about +2 percentage points in acc. and F1 compared with the best baseline, RoBERTa-base.

- Empathy cue extraction task (identifying empathy-related words/phrases in replies):

**Baselines:** TF–IDF + logistic regression, GPT-2, DialoGPT;

**Metrics:** token-level t-acc., t-f1, and IOU-f1.

**Result:** the Empathy Computing Model achieves higher scores than the strongest baseline on all three metrics, with particularly notable gains in IOU-f1. This suggests that the dual-encoder plus attention mechanism helps the model locate empathy cues more accurately in context.

**Qualitative error analysis indicates two typical failure cases:**

Long replies with sparse empathy markers:

When a long reply contains only a few short but strong expressions of empathy, the model sometimes underestimates the overall empathy level.

Rapid topic shifts:

When the reply quickly shifts from emotional processing to factual discussion, the model may mis-classify the dominant ESC stage or assign an incorrect empathy dimension.

These limitations point to directions for future refinement of the empathy computing model.
